# Tandem malonate-based glucosides (TMGs) for membrane protein structural studies

**DOI:** 10.1038/s41598-017-03809-3

**Published:** 2017-06-21

**Authors:** Hazrat Hussain, Jonas S. Mortensen, Yang Du, Claudia Santillan, Orquidea Ribeiro, Juyeon Go, Parameswaran Hariharan, Claus J. Loland, Lan Guan, Brian K. Kobilka, Bernadette Byrne, Pil Seok Chae

**Affiliations:** 1 Department of Bionanotechnology, Hanyang University, Ansan, 155-88 South Korea; 2 Center of Neuroscience, University of Copenhagen, Copenhagen, DK-2200 Denmark; 3 Molecular and Cellular Physiology, Stanford University, Stanford, CA 94305 USA; 4 Department of Cell Physiology and Molecular Biophysics, Center for Membrane Protein Research, School of Medicine, Texas Tech University Health Sciences Center, Lubbock, TX 79430 USA; 5 Department of Life Sciences, Imperial College London, London, SW7 2AZ UK

## Abstract

High-resolution membrane protein structures are essential for understanding the molecular basis of diverse biological events and important in drug development. Detergents are usually used to extract these bio-macromolecules from the membranes and maintain them in a soluble and stable state in aqueous solutions for downstream characterization. However, many eukaryotic membrane proteins solubilized in conventional detergents tend to undergo structural degradation, necessitating the development of new amphiphilic agents with enhanced properties. In this study, we designed and synthesized a novel class of glucoside amphiphiles, designated tandem malonate-based glucosides (TMGs). A few TMG agents proved effective at both stabilizing a range of membrane proteins and extracting proteins from the membrane environment. These favourable characteristics, along with synthetic convenience, indicate that these agents have potential in membrane protein research.

## Introduction

Membrane proteins constitute approximately one third of the total proteome of all organisms^1^ and they are the targets of most currently available drugs^2^. However, less than 1% of all membrane proteins have been structurally characterized^3^, limiting understanding of their precise molecular mechanisms of action and slowing progress in protein structure-based rational drug design. The major hurdle in structural determination arises mainly from the instability of membrane proteins in aqueous solution. Membrane proteins are remarkably stable when inserted into the native membranes, but biophysical methods such as X-ray crystallography and nuclear magnetic resonance (NMR) spectroscopy, widely used for protein structural characterization are incompatible with these membrane systems^4^. Detergents are the most-widely used tools for membrane protein extraction from the native membranes. Due to their amphipathic nature, detergent micelles are capable of effectively interacting with lipid bilayers as well as membrane proteins, resulting in the disruption of lipid bilayers and the formation of protein–detergent complexes (PDCs). More than 120 conventional detergents are available, but non-ionic detergents such as OG (*n*-octyl-β-d-glucoside), DM (*n*-decyl-β-d-maltoside) and DDM (*n*-dodecyl-β-d-maltoside) are most widely used for the structural characterizations of membrane proteins^5–10^. However, many membrane proteins, particularly complexes, solubilized even in these popular detergents have the tendency to denature/aggregate over the course of sample preparation for downstream characterization^11, 12^. In contrast to the large diversity in the function and 3D structures of membrane proteins, conventional detergents typically bear a single flexible alkyl chain and a single head group, thus significantly restricting their properties^11, 12^. Thus, it is of tremendous interest to develop new amphiphilic agents with enhanced efficacy toward many membrane proteins recalcitrant to structural analyses in conventional detergents^12, 13^.

**Figure 1:**
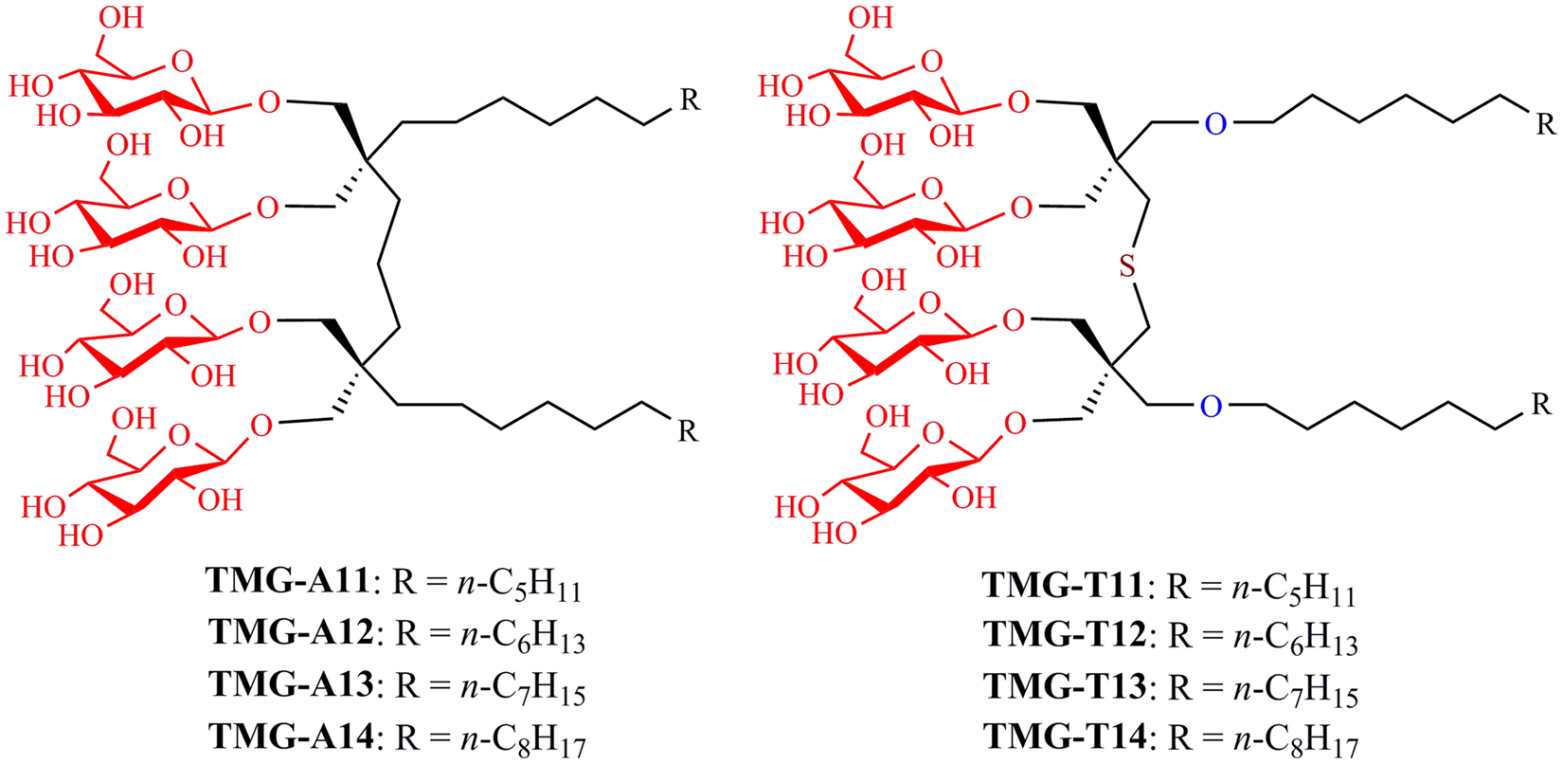
Chemical structures of the tandem malonate glucosides (TMGs). Two malonate units were connected via a propylene linker in the case of the TMG-As or *via* a dimethyl sulfide linker for the TMG-Ts. The alkyl chain length varied from C11 to C14 for both sets of TMGs, and this was incorporated into the detergent designation.

A number of novel agents with non-traditional architecture have been developed to expand on the narrow range of detergent properties. Representatives include small amphiphilic molecules such as tripod amphiphiles (TPAs)^12, 14–16^, facial amphiphiles (FAs)^17, 18^, glyco-diosgenin (GDN)^19^ and neopentyl glycol (NG) amphiphiles (NDTs, GNGs and MNGs)^20–22^, mannitol-based amphiphiles (MNAs)^23^, and penta-saccharide-based amphiphiles (PSEs)^24^. In addition, oligomeric/polymeric materials such as amphipols^25^, lipopeptide detergents (LPDs)^26^, and β-peptide (BPs)^27^ were developed as alternatives to small amphiphilic molecules. Some of these membrane-mimetic systems contain a patch of lipid bilayer stabilized by surrounding amphipathic agents, as exemplified by bicelles and nanodiscs (NDs)^28, 29^. Despite their excellent efficacy toward protein stabilization, most of these large membrane-mimetic systems (e.g., amphipols and NDs) are not efficient at extracting proteins from the membranes, or have yet to produce high quality protein crystals. Small amphiphilic molecules tend to be more effective at extracting proteins from the membranes, but they are not usually as effective as the large membrane-mimetic systems at stabilizing membrane protein structures^29^. In addition, small glucoside detergents have been demonstrated to be inferior to their maltoside counterparts with respect to protein stabilization (e.g., OG *vs* DDM), but may be more suitable for crystallisation presumably due to the small size of the micelle^11, 20^. Thus, it is particularly challenging to develop small glucoside detergents with enhanced protein-stabilizing efficacy relative to DDM, the gold standard conventional detergent. In the present study, we designed and synthesized novel glucosides by connecting two malonate-based core units *via* an alkyl or thioether linkage, designated alkyl chain- or thioether-linked tandem malonate-based glucosides (TMG-As/Ts) (Fig. 1). When these agents were evaluated for multiple membrane protein systems, TMG representatives conferred enhanced stability to most of the tested proteins compared to DDM, with the best detergent variable depending on the target protein.

## Results

### Design, synthesis and physical properties of TMG amphiphiles

The newly designed amphiphiles feature two alkyl chains and two branched diglucosides as tail and head groups, respectively (Fig. 1). These agents are structurally distinct from GNGs that we developed previously^21^. Both TMGs and GNGs share a central malonate-based unit, but the GNGs contain a single malonate-derived unit while the TMGs have two of these units linked by a short alkyl chain.^[11]^ This difference results in variation in detergent inter-alkyl chain distance, the number of glucoside units, detergent geometry and detergent flexibility. The TMGs were divided into two groups according to the linker structure: TMG-As and TMG-Ts (Fig. 1). The TMG-As contain two malonate-derived units connected to each other *via* a propylene linker, different from the TMG-Ts with a thioether-functionalized linker (dimethyl sulfide linker). In addition, the two alkyl chains were introduced into the tandem malonate-based core *via* ether linkages (TMG-Ts) or directly (TMG-As). Since the optimal balance between hydrophilicity and hydrophobicity is known to be essential for effective stabilization of membrane proteins^30^, detergent alkyl chain length was also varied from C11 to C14. Both sets of the novel agents (TMG-As/Ts) were prepared using a straightforward synthetic protocol. The TMG-As were synthesized in five steps: alkyl connection of two malonate units, dialkylation and reduction of tetra-ester derivatives, glycosylation and global deprotection (see Supplementary scheme 1). The same number of synthetic steps was necessary for the preparation of the TMG-Ts (see Supplementary scheme 2). In this case, however, the initial three synthetic steps could be combined together to obtain the dialkylated tetra-ol derivatives in a single chromatographic separation, thus increasing synthetic accessibility of the TMG-Ts. The final agents could be prepared at an overall yield of more than 30% for both TMG sets without an optimization process (see Supplementary synthetic protocol). The convenient synthesis and high synthetic yield make preparation of these agents feasible in multi-gram quantity.

All the TMGs were water-soluble up to 10% and micelles formed by these agents were stable enough to give clear solutions for several months. These novel agents were physically characterized in terms of critical micelle concentrations (CMC) and the hydrodynamic radii (*R*_h_) of their micelles. The CMCs were estimated using a hydrophobic fluorophore^31^ while the hydrodynamic radii (*R*_h_) were measured using dynamic light scattering (DLS). The summarized results for both sets of the TMGs along with a conventional detergent (DDM) are presented in Table 1. The CMC values of the novel agents varied from 4 µM to 20 µM, all substantially smaller than DDM (170 µM), indicating that the TMGs have a 10–40 times higher tendency to self-aggregate than DDM. The CMC values of the TMGs decreased with increasing alkyl chain length, consistent with the general idea that hydrophobicity of the alkyl chain is the main factor in determining detergent CMC. For instance, TMG-A14, with the longest alkyl chain, gave a CMC value four times smaller than TMG-A11, with the shortest alkyl chain. No major difference was observed in the CMC values between the TMG-As and the equivalent TMG-Ts although the CMCs for the TMG-Ts were slightly higher than the TMG-As in this respect. The sizes of micelles formed by the TMGs increased from 3.1/3.0 to 3.8 nm with increasing alkyl chain length from C11 to C14, more or less comparable to that formed by DDM (3.4 nm; C12). The TMG-Ts tend to form smaller micelles than the TMG-As (TMG-T12 (3.1 nm) *vs* TMG-A12 (3.3 nm)). When DLS data were further analysed with regard to number-averaged size distributions, all of these detergents (TMG-As and TMG-Ts) produced a single set of micelle populations, indicative of high micellar homogeneity (see Supplementary Fig. 1).

**Table 1:** Molecular weights and critical micelle concentrations (CMCs) of TMGs (TMG-As/Ts) and a conventional detergent (DDM) and hydrodynamic radii (*R*_h_; *n* = 5) of their micelles measured in pure water.

Detergent	*M.W*.^a^	CMC (mM)	CMC (wt%)	*R*_h_ (nm)b
TMG-A11	1149.41	~0.015	~0.0017	3.1 ± 0.15
TMG-A12	1177.47	~0.010	~0.0012	3.3 ± 0.09
TMG-A13	1205.52	~0.006	~0.0007	3.6 ± 0.16
TMG-A14	1233.58	~0.004	~0.0005	3.8 ± 0.10
TMG-T11	1227.50	~0.020	~0.0025	3.0 ± 0.07
TMG-T12	1255.55	~0.015	~0.0019	3.1 ± 0.06
TMG-T13	1283.61	~0.006	~0.0008	3.3 ± 0.08
TMG-T14	1311.66	~0.004	~0.0005	3.8 ± 0.09
DDM	510.1	~0.17	~0.0087	3.4 ± 0.03

^a^Molecular weight of amphiphiles. ^b^Hydrodynamic radius of detergent micelles determined at 1.0 wt% by dynamic light scattering.

### TMG evaluation with a diverse set of membrane proteins

**Figure 2:**
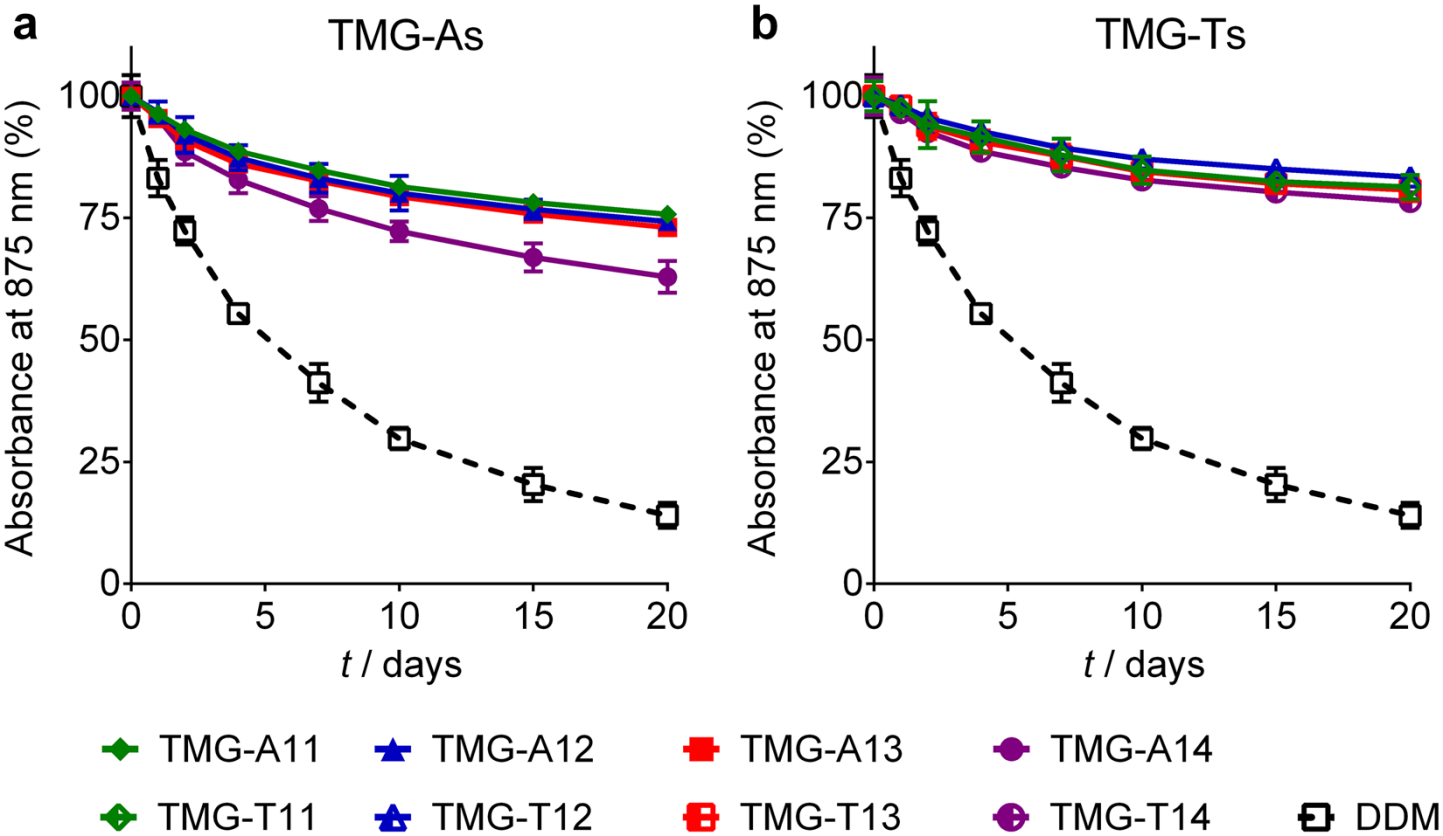
Long-term stability of *R. capsulatus* superassembly solubilized in the TMG-As (TMG-A11, TMG-A12, TMG-A13 and TMG-A14) (**a**) or the TMG-Ts (TMG-T11, TMG-T12, TMG-T13 and TMG-T14) (**b**). A conventional detergent (DDM) was used as a control. All the detergents were used at CMC + 0.2 wt%. Protein stability was monitored by measuring absorbance value at 875 nm (A_875_) at regular intervals over a 20 day-incubation at 25 °C. Error bars, SEM, *n* = 2.

The newly prepared TMGs were first evaluated using the *Rhodobacter (R.) capsulatus* super-assembly, comprising light harvesting complex I and the reaction centre complex (LHI-RC)^32^. This complex is known to be particularly sensitive to protein denaturation and this denaturation can be readily assessed by measuring the absorbance at 875 nm which detects the presence of cofactors (e.g., chlorophylls and carotenoids) embedded in the folded state^33^. For detergent evaluation, DDM-purified LHI-RC complex (80 μL) was diluted into buffer solutions (920 μL) containing individual detergents (TMG-As, TMG-Ts or DDM) to give final protein and detergent concentrations of 0.2 μM and CMC + 0.2 wt%, respectively. The residual amount of DDM is estimated to be ~0.005 wt%, assuming that a single membrane protein is surrounded by 400 DDM molecules. It was reported that the aggregation number of DDM in association with membrane proteins vary in a typical range of 100 to 400, depending on target proteins^34^. Thus, the estimated residual amount of DDM was considered negligible compared to that of the novel agents (0.005 *vs* 0.2 wt%). Protein stability was assessed by measuring protein absorbance at 875 nm (A_875_) at regular intervals over the course of a 20-day incubation at room temperature. As can be seen in Fig. 2, all of the TMGs were clearly superior to DDM at preserving complex integrity. When the two groups of TMGs were compared, the TMG-Ts were a little better than the TMG-As. TMG-A14 was the worst novel agent, but still significantly better than DDM in this regard. When detergent concentration was decreased to CMC + 0.04 wt%, similar trends were observed although the efficacy difference between the TMGs and DDM was reduced (see Supplementary Fig. 2). It is notable that all the TMGs were effective at stabilizing the complexes at both tested detergent concentrations whereas DDM efficacy was significantly decreased at the higher concentration of CMC + 0.2 wt%.

**Figure 3:**
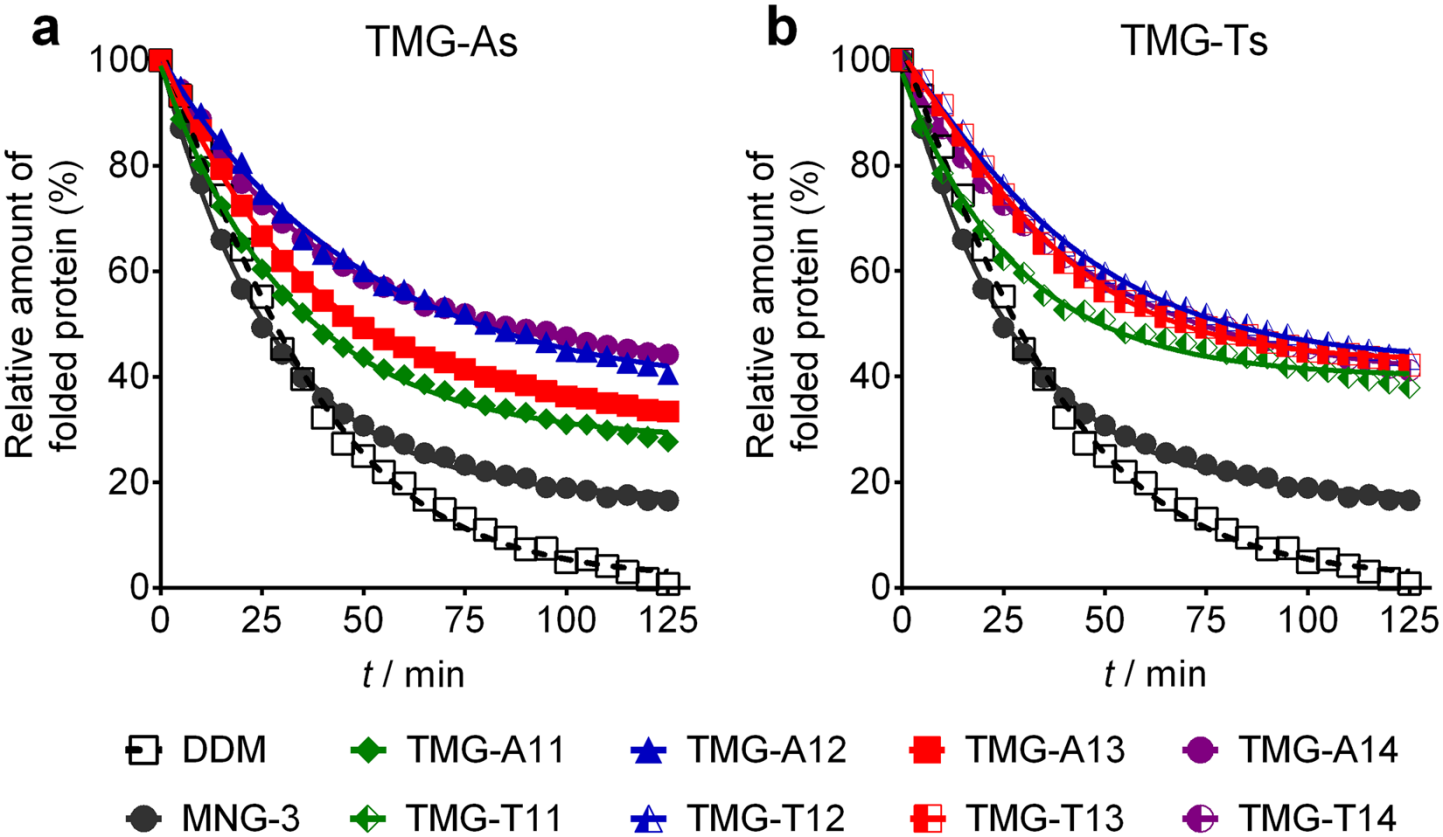
Thermal denaturation profile of UapA solubilized in individual agents (TMG-As (**a**) and TMG-Ts (**b**), MNG-3, or DDM) used at CMC + 0.04 wt%. The relative amounts of the folded transporter were monitored using CPM assay carried out at 40 °C for 125 min. Mean standard deviation (*n* = 3) for DDM, TMG-A11, TMG-A12, TMG-A13, TMG-A14, MNG-3, TMG-T11, TMG-T12, TMG-T13 and TMG-T14 are 4.8, 5.2, 7.6, 3.4, 7.3, 5.1, 6.1, 4.6, 7.2, and 8.8, respectively.

The novel agents were further evaluated with the uric acid-xanthine/H^+^ symporter (UapA) from *Aspergillus nidulans* ^35^. UapA has been structurally characterised and shown to exist as a closely associated dimer in DDM^36^. In order to assess protein stability, a thermal denaturation assay was carried out using a sulfhydryl-specific fluorophore, *N*-[4-(7-diethylamino-4-methyl-3-coumarinyl)phenyl]maleimide (CPM)^37^. As fluorescence emission intensity increases with the amounts of unfolded proteins in samples, CPM serves as a protein unfolding sensor. For this assay, DDM-purified transporter was diluted 1:150 into buffer solutions containing individual amphiphiles (TMG-As, TMG-Ts, or DDM) to give final protein and detergent concentrations of 0.2 μM and CMC + 0.04 wt%, respectively. Assuming that 400 DDM molecules aggregated around a single UapA dimer, DDM concentration is estimated to become ~0.011 wt% after sample dilution, lower than that of the novel agents (CMC + 0.04 wt%). The changes in fluorescence intensity of the samples were monitored regularly during a 125-min incubation at 40 °C. All of the novel agents (TMGs) were significantly better than DDM at preserving the transporter in the folded state (Fig. 3). Again, the TMG-Ts appeared to behave slightly better than the TMG-As. Of all tested TMGs, the shortest alkyl chain TMGs (TMG-A11/T11) were the least effective. The suboptimal property of these C11 alkyl chain agents was further demonstrated when the detergents were used at CMC + 0.2 wt%. At this concentration, TMG-A11 and TMG-T11 were worse than and just comparable to DDM, respectively. The TMG-Ts are generally better than the TMG-As at maintaining the folded state of the transporter, with TMG-A14 and TMG-T13 being the best performing agents of the TMG-As and TMG-Ts, respectively (see Supplementary Fig. 3). This result suggests that the long alkyl chain TMGs (e.g., TMG-T13/A14) are more favourable than the short alkyl chain counterparts (e.g., TMG-T11/A11) at stabilizing the transporter. These long alkyl chain TMGs were better than MNG-3 (commercial name: LMNG), a widely used novel agent, at stabilizing the transporter. MNG-3 was only marginally better than DDM for this protein under the conditions tested (Fig. 3 and Supplementary Fig. 3).

**Figure 4:**
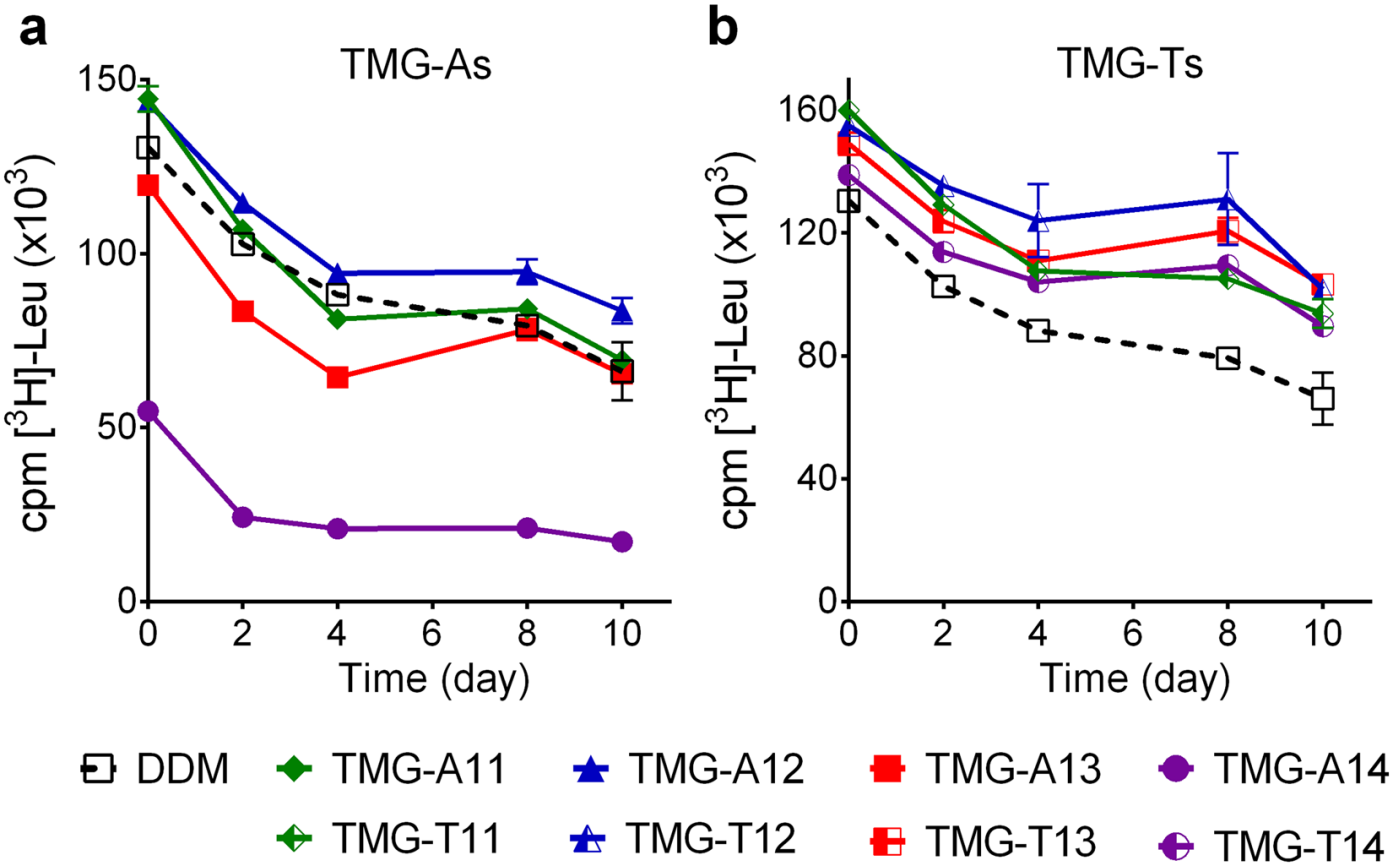
Long-term activity of LeuT solubilized in the TMG-As (TMG-A11, TMG-A12, TMG-A13, or TMG-A14) (**a**) or TMG-Ts (TMG-T11, TMG-T12, TMG-T13, or TMG-T14) (**b**). Detergent efficacy of the TMGs was compared with DDM, a gold standard conventional detergent. LeuT stability was assessed by measuring the ability to bind a radiolabeled leucine ([^3^H]-Leu) *via* scintillation proximity assay (SPA) and monitored at regular intervals over the course of a 10-day incubation at room temperature. The results are expressed as specific binding of [^3^H]-Leu (mean ± SEM, *n* = 2). All detergents were used at CMC + 0.04 wt%.

The new detergents were further tested with the bacterial leucine transporter (LeuT) from *Aquifex aeolicus* ^38^. To start with, DDM-purified transporter (100 μL) was mixed with individual detergent-containing solutions (900 μL) to give final protein and detergent concentration of 0.2 μM and CMC + 0.04 wt%, respectively. Following the sample dilution, the residual amount of DDM is calculated to be ~0.030 wt% using the aggregation number of DDM (i.e., 226) specifically reported for LeuT^39^, lower than the concentration of the novel agents (CMC + 0.04 wt%). Protein stability was assessed by measuring the ability of the transporter to bind a radiolabeled substrate ([^3^H]-leucine) using scintillation proximity assay (SPA)^40^. The substrate binding activity of the transporter was monitored at regular intervals during an incubation period of 10 days at room temperature (Fig. 4a). At this low detergent concentration, the stability of the protein in the TMG-As varied substantially depending on the alkyl chain length; the TMG-As with a shorter chain (e.g., TMG-A11/C12) were comparable to DDM while TMG-A14 with the longest alkyl chain was the least stabilizing. TMG-A13 with one carbon unit shorter than TMG-A14 was a little worse than DDM. A similar result was obtained when detergent concentration was increased to CMC + 0.2 wt% (see Supplementary Fig. S4a). Again, TMG-A12 was the most stabilizing detergent of the TMG-As, followed by TMG-A11 and TMG-A13. The longest alkyl chain TMG (TMG-A14) was again the least stabilizing. At CMC + 0.04 wt%, all TMG-Ts were markedly better at retaining the activity of the transporter than both DDM and the TMG-As. The best performing agent was TMG-T12 (Fig. 4b). When detergent concentration was increased to CMC + 0.2 wt%, all TMG-Ts except TMG-T14 were better than DDM at retaining activity of the transporter (see Supplementary Fig. S4b). Based on these results, the C12 alkyl chain in the TMG architecture appeared to be optimal for transporter stability. Finally, in the absence of the TMGs (i.e., detergent-free condition), the ability of LeuT to bind the radiolabeled substrate was reduced to 25% of that of DDM by a 30-min incubation (see Supplementary Fig. S5). A further decrease in transporter activity was observed in the course of a 20-hour incubation. This result indicates that the estimated residual DDM (~0.030 wt%), although present at a higher concentration than the CMC (~0.0087 wt%), is not enough to preserve stability of the transporter. Thus, the presence of the individual TMGs appears to be essential for transporter stability.

**Figure 5:**
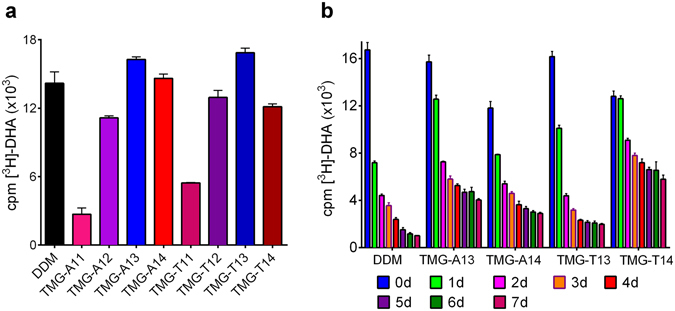
Ligand binding activity for β_2_AR solubilized in DDM or TMGs. The DDM-purified β_2_AR stock was 500-fold diluted in CMC + 0.2% DDM or TMGs (TMG-As or TMG-Ts). (**a**) Activity of DDM- or TMG-solubilized receptor was measured following 30-min incubation by radiolabeled ligand binding assay using the antagonist [^3^H]-DHA. (**b**) Receptor functionality was additionally assessed in the best performing detergents identified in (**a**) over a period of 7 days with samples taken for analysis every 24 hours. Error bars, SEM, *n* = 3.

The intriguing results of the TMGs encouraged us to test these agents with the human β_2_ adrenergic receptor (β_2_AR), a G-protein coupled receptor (GPCR)^41^. The receptor stability was assessed *via* a ligand binding assay using the antagonist, [^3^H]-dihydroalprenolol ([^3^H]-DHA)^42^. The assay started with the 150-fold dilution of DDM-purified receptor into detergent solutions containing either DDM or individual TMGs (TMG-As and TMG-Ts) to reach final protein and detergent concentrations of 0.2 μM and CMC + 0.2 wt%, respectively. The residual DDM concentration, which is estimated to be ~0.0007 wt% by assuming 400 DDM molecules/receptor, was negligible compared to the final concentration of a novel agent (~0.2 wt%). Following a 30-min incubation to allow for complete detergent exchange, the ligand binding activity of the receptor was monitored. Some TMGs such as TMG-A13/A14 and TMG-T13/T14 were as effective as DDM at retaining receptor activity (Fig. 5a). Thus, these agents were selected for further analysis where receptor activity was monitored regularly over the course of 7-day incubation at room temperature. In this experiment, TMG-A13 and TMG-T13 were superior to DDM at maintaining receptor activity long-term, with TMG-A13 outperforming TMG-T13 (Fig. 5b). Similarly, TMG-A14 and TMG-T14 were superior to DDM although these agents were a little worse than DDM in terms of initial receptor activity (*t* = 0). Of the TMGs tested here, TMG-T14 was best at preserving receptor activity, followed by TMG-A13 and TMG-A14. When the DDM-solubilized receptor was diluted into TMG-free buffer solution (i.e., detergent-free condition), the receptor rapidly degraded over time, giving only ~10% residual protein activity after a 3-hour incubation (see Supplementary Fig. S6). This result indicates that the receptor cannot maintain structural and functional integrity in the absence of the individual TMGs. We selected two TMGs (TMG-A13 and TMG-T14) to further investigate these agents in terms of homogeneity of receptor-detergent complexes. A SEC result showed that each TMG produced monodisperse complexes with β_2_AR, similar to that formed by DDM (see Supplementary Fig. S7). This result implies that TMG-A13 and TMG-T14 may hold significant potential for GPCR study.

For each of the membrane proteins tested above, a conventional detergent (DDM) was first used to solubilize and purify the target protein and this DDM-purified protein was then diluted into individual detergent-containing solutions. Thus, mixed detergent systems containing small amounts of residual DDM were used for detergent efficacy comparison. As for LHI-RC and β_2_AR, the residual amounts of DDM (<0.005 wt%) were much smaller than those of the individual novel detergents (i.e., CMC + 0.04/0.2 wt%) and even smaller than CMC value of DDM (~0.0087 wt%). As for two of the transporters (UapA and LeuT), the residual DDM amounts are estimated to be 0.011 and 0.030 wt%, respectively. These concentrations are comparable to the low concentrations of the TMGs (CMC + 0.04 wt%), but are substantially smaller than the high TMG concentrations (CMC + 0.2 wt%). As observed in the detergent-free condition, the residual amount of DDM (~0.030 wt%) was too small to maintain LeuT activity. In the case of UapA, as we have used the highest predicted aggregation number for DDM associated with the protein (400 molecules of DDM/UapA dimer), it is possible that in reality this is lower. Furthermore, the effect of residual DDM on protein stability should be similar from one sample to another as detergent evaluation was carried out in each case using a side-by-side comparison. Thus, the residual amount of DDM is unlikely to interfere with evaluation of the novel agents for the membrane proteins studied here. However, we can’t completely exclude that residual DDM is having an effect on our analysis of these proteins.

**Figure 6:**
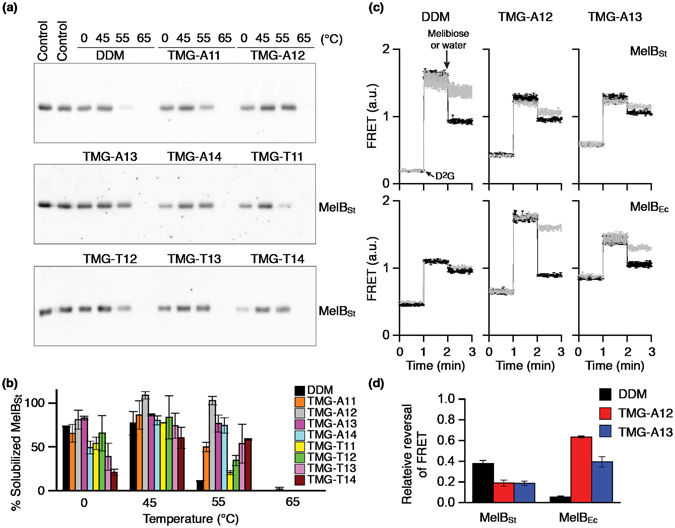
Thermostability and functionality of MelB_St_ solubilized in DDM or individual novel agents (TMG-As: TMG-A11, TMG-A12, TMG-A13 and TMG-A14; TMG-Ts: TMG-T11, TMG-T12, TMG-T13 and TMG-T14). *E. coli* membranes containing MelB_St_ were mixed with the indicated detergent, and then kept at 0 °C or an elevated temperature (45, 55, or 65 °C) for 90 minutes. (**a**) Western blott analysis. The amount of soluble MelB_St_ after ultracentrifugation was detected by penta-His-HRP antibody. The protein samples were initially separated on SDS-15%PAGE gels. (**b**) Histogram. The density representing the soluble MelB_St_ in individual detergents detected in panel (**a**) was measured by ImageQuant software and expressed as a percentage (%) relative to that present in the untreated membrane sample (**b**). Error bars, SEM, *n* = 3. (**c**) MelB Trp→ D^2^G FRET reversal functional asssay. Sample preparations and FRET measurements are described in the Methods. The FRET signals were monitored over time. D^2^G at 10 μM was added at the 1-min time point and melibiose (black trace) at a saturating concentration added at the 2-min time point. Control experiments were carried out by adding water (gray trace) instead of melibiose at the 2-min time point. (**d**) Relative values for FRET reversal were obtained by calculating fluorescent intensity decrease (at 2-min point)/increase (at 1-min point).

In order to assess this further, all the TMG agents were used to extract the *Salmonella typhimurium* melibiose permease (MelB_St_) directly from *E. coli* membranes^43, 44^. The membrane fractions were mixed with 1.5% DDM or individual TMG detergents (TMG-As or TMG-Ts) on ice and the resulting solutions were then incubated for 90 min at four different temperatures (0, 45, 55, and 65 °C). The amount of MelB_St_ extracted and stabilised by each detergent was analyzed through SDS-PAGE and Western blotting after separation by ultracentrifugation (Fig. 6a), and expressed as a percentage of the total amount of MelB_St_ initially present in the untreated membrane (Fig. 6b). At 0 °C, the amounts of soluble MelB_St_ were smaller than DDM for all of the TMGs except TMG-A12 and TMG-A13. The two novel agents (TMG-A12 and TMG-A13) were as efficient as DDM at extracting MelB_St_. When heating the samples at 45 °C, however, all TMGs except TMG-T14 were comparable to DDM at maintaining MelB_St_ in solution. Notably, TMG-A12 gave full retention of soluble MelB_St_ at this temperature and even at 55 °C. In contrast, DDM gave only ~10% soluble MelB_St_ at 55 °C. Incubation at 65 °C resulted in a complete loss of MelB_St_ from the solutions in all cases. The well-behaving TMGs (TMG-A12 and TMG-A13) were further evaluated in terms of MelB function monitored by measuring FRET from tryptophan residues to 2′-(*N*-dansyl)aminoalkyl-1-thio-β-d-galactopyranoside (D^2^G) bound to the protein (i.e., Trp→ D^2^G FRET)^45^. Upon addition of D^2^G, a functional MelB_St_ gives an increase in fluorescence intensity induced by Trp→ D^2^G FRET that can be reversed by adding a non-fluorescent sugar substrate (i.e., melibiose). Upon addition of melibiose, the DDM-solubilized MelB_St_ gave a large reversal in the FRET signal while the TMG-A12 or TMG-A13-solubilized MelB_St_ appeared to be less responsive in this regard (Fig. 6c and d). A similar trend was observed for MNG-3-solubilized MelB_St_ in a previous study^46^. When we used MelB from *Escherichia coli* (MelB_Ec_), known to be less stable than MelB_St_^46^, DDM failed to give functional protein. In contrast, TMG-A12 or TMG-A13 resulted in a functional MelB_Ec_ as demonstrated by large changes in FRET signal. These results indicate that these novel agents, particularly TMG-A12, are effective at maintaining MelB functionality as well as solubility.

## Discussion

Detergent efficacy can be considerably affected by a minor change in detergent structure. Despite the small variations in the chemical structures, the different TMGs showed marked variations in membrane protein stabilization. The TMG-Ts were overall better than the TMG-As at stabilizing all the tested membrane proteins except MelB_St_. In addition, the best detergents varied depending on the individual target proteins. TMG-T12 and TMG-T13 were best for LeuT and UapA stability, respectively, while TMG-A13/TMG-T14 and TMG-A12 were best for β_2_AR and MelB_St_, respectively. It is notable that short alkyl chain TMGs (e.g., TMG-A11/A12 and TMG-T11/T12) tended to be favorable for LeuT stability while long alkyl chain TMGs (e.g., TMG-A13/A14 and TMG-T13/T14) were generally advantageous for β_2_AR and UapA stability. Of the TMG-As and TMG-Ts, TMG-A12/TMG-A13 and TMG-T13/TMG-T14 were the best overall at maintaining protein stability; these agents were superior or at least comparable to DDM in maintaining the stability of all the tested proteins. The current paradigm is that the most suitable detergent tends to be protein-specific. Because there is a large diversity in the tendency of membrane proteins to aggregate/denature and a substantial variation in protein dimensions, it is unlikely that a single or even a small number of detergents can act as a magic bullet for all or most membrane proteins. However, our result indicates that it may be possible to develop a detergent suitable for a large number of membrane proteins. Detergent efficacy also tended to depend on detergent concentration. In the current study, detergent concentration of CMC + 0.04 or 0.2 wt% was used for detergent evaluation with LHI-RC, UapA and LeuT. As for LHI-RC and LeuT, the novel agents showed reduced protein stability at a high concentration (CMC + 0.2 wt%). A similar trend was observed for UapA, although it is less clear. This result is consistent with the notion that excess detergent micelles produced by increased detergent concentration is detrimental for protein stability^47^. Note that an optimal detergent concentration could significantly vary depending on the property and concentration of a target membrane protein.

Currently, there are a very limited number of glucoside detergents that confer consistently greater stability to a range of membrane proteins than DDM; OG is known to be significantly less effective at stabilizing membrane proteins than DDM^11^ and the most widely used novel glucoside agent, GNG-3 (commercial name: OGNG), was shown to be worse than DDM in terms of stabilizing a range of membrane proteins^21^. However, due in part to the presence of the comparatively small hydrophilic group, OG is widely used for membrane protein crystallization. In addition, GNG-3 has contributed to the crystal structure determination of several membrane proteins in the last four years^48–51^. Thus, it is of great importance to develop additional glucoside detergents with enhanced protein stability compared to DDM or GNG-3. Achieving enhanced stability for multiple membrane proteins is challenging as detergent efficacy tends to be protein-specific as described above. However, on the basis of our previous and current studies^21^, we seem to have achieved this with at least some of the TMGs (e.g., TMG-A12/A13 and TMG-T13/T14) as these agents were shown to be more effective than both DDM and GNG-3 at stabilizing a range of membrane proteins.

In summary, two groups of novel glucoside agents were prepared by connecting two malonate-based units with a propylene or dimethyl sulfide spacer. When these TMG agents were evaluated with five membrane proteins in terms of protein stabilization, the TMG-Ts were generally superior to the TMG-As for three membrane protein systems (LHI-RC complex, UapA and LeuT). An opposite trend was observed for MelB_St_ stability while the comparable efficacy of the TMGs was found for β_2_AR. More importantly, some of the TMGs (TMG-A12/TMG-A13 and TMG-T13/TMG-T14) were more effective than or at least comparable to DDM at maintaining the stability of the tested membrane proteins here, suggesting wide applicability of these agents. As glucoside detergents are advantageous for membrane protein structural study, these synthetically accessible TMG agents have potential for membrane protein research.

## Methods

### *R. capsulatus* superassembly stability assay

The *R. capsulatus* superassembly expressed in the engineered strain of *Rhodobacter capsulatus* was solubilized and purified according to the reported protocol^33^. A 10 mL aliquot of the frozen membranes was thawed and homogenized using a glass tissue homogenizer at room temperature. The homogenate was incubated with mild agitation at 32 °C for 30 min. After the addition of 1.0 wt% DDM, the homogenate was incubated for an additional 30 min at 32 °C. Following ultracentrifugation, the supernatant containing the solubilized LHI-RC complexes was collected and incubated with Ni^2+^-NTA resin at 4 °C for one hour. The resin was loaded into 10 His-SpinTrap columns separately and washed twice with 500 μL binding buffer (10 mM Tris (pH 7.8), 100 mL NaCl, 1 × CMC DDM). A binding buffer containing 1 M imidazole (2 × 300 μl) was used to elute DDM-purified LHI-RC complex. 80 μL of the DDM-purified LHI-RC complex was diluted into 920 μL of individual detergent solutions; TMG-As (TMG-A11, TMG-A12, TMG-A13 and TMG-A14), TMG-Ts (TMG-T11, TMG-T12, TMG-T13 and TMG-T14) or DDM to reach a final detergent concentration at CMC + 0.04 wt% or CMC + 0.2 wt%. Sample dilution was carried out for one hour and the complex was incubated at room temperature for 20 days. Protein stability was measured at regular intervals during the incubation by measuring UV-Visible spectra of the samples in the range of 650 to 950 nm.

### UapA thermal denaturation assay

UapAG411V_Δ1–11_ from *Aspergillus nidulans* was expressed as a C-terminal GFP fusion protein in the FGY217 strain of *Saccharomyces cerevisiae*. The UapA was isolated and purified in sample buffer (20 mM Tris (pH 7.5), 150 mM NaCl, 0.03% DDM, 1 mM xanthine) according to the reported protocol^52^. The protein was concentrated to approximately 10 mg/mL using a 100 kDa molecular weight cut off filter (Millipore). The protein was diluted 1:150 into buffer containing either TMG-As (TMG-A11, TMG-A12, TMG-A13 and TMG-A14), TMG-Ts (TMG-T11, TMG-T12, TMG-T13 and TMG-T14) or DDM to give final detergent concentrations of CMC + 0.04 wt% or CMC + 0.2 wt% in Greiner 96-well plates. The CPM dye (Invitrogen) stored in DMSO (Sigma) was diluted in dye buffer (20 mM Tris (pH 7.5), 150 mM NaCl, 0.03% DDM, 5 mM EDTA) and 3 μL of the dye buffer was added to each sample. Protein stability was measured by incubating the reaction mixture for 125 min at 40 °C, starting from 30 min after sample dilution. The fluorescence emission was recorded using a microplate spectrofluorometer set at excitation and emission wavelengths of 387 nm and 463 nm, respectively. The relative amounts of folded proteins were plotted against time using GraphPad Prism.

### LeuT stability assay

Leucine transporter (LeuT) from *Aquifex aeolicus* was expressed in *E. coli*, C41 (DE3) cells transformed with pET16b encoding the 8xHis-tagged transporter. LeuT was extracted and purified according to the reported protocol^38^. The isolated bacterial transporter was solubilized in 1.0 wt % DDM. The DDM-solubilised protein was bound to Ni^2+^-NTA resin (Life Technologies, Denmark) and was eluted with elution buffer containing 20 mM Tris-HCl (pH 8.0), 1 mM NaCl, 199 mM KCl, 0.05% DDM and 300 mM imidazole. Finally, 1.5 mg/mL protein stock was diluted in identical buffer without DDM and imidazole, but supplemented with individual TMG-As (TMG-A11, TMG-A12, TMG-A13 and TMG-A14), TMG-Ts (TMG-T11, TMG-T12, TMG-T13 and TMG-T14) or DDM (a positive control) to reach a final concentration of CMC + 0.04 wt% or CMC + 0.2 wt%. As a negative control, the protein stock was diluted into a detergent-free buffer solution. The samples stood for one hour to allow detergent exchange and were then stored for 10 days at room temperature, centrifuged at the indicated time points and the ligand binding activity was measured using [^3^H]-Leu via scintillation proximity assay (SPA)^40^. SPA was performed at the above-mentioned detergent concentrations with 5 μL of the respective protein samples, 20 nM [^3^H]-Leu and 1.25 mg/mL copper chelate (His-Tag) YSi beads (both from Perkin Elmer, Denmark) in buffer containing 450 mM NaCl. [^3^H]-Leu binding was determined via MicroBeta liquid scintillation counter (Perkin Elmer).

### β_2_AR long-term stability assay

β_2_AR was isolated and purified in 0.1% DDM according to the reported protocol^42^. Briefly, β_2_AR was expressed in Sf9 insect cells infected with baculovirus and solubilized in 1% DDM. The DDM-purified β_2_AR was added to individual TMG-containing buffers (TMG-As (TMG-A11, TMG-A12, TMG-A13 and TMG-A14), TMG-Ts (TMG-T11, TMG-T12, TMG-T13 and TMG-T14), GNGs (GNG-2 and GNG-3), or DDM to make a final concentration at CMC + 0.2 wt%. As a control, the DDM-purified β_2_AR was diluted into a detergent-free buffer. After allowing 30-min sample dilution, β_2_AR solubilized in individual detergents was stored for 6 or 7 days at room temperature and ligand binding ability was assessed at regular intervals over this period by incubating the samples with 10 nM [^3^H]-dihydroalprenolol (DHA) supplemented with 0.5 mg/ml BSA for 30 min at room temperature. The combined mixture was loaded onto a G-50 column and the flowthrough was collected in 1 ml binding buffer (20 mM HEPES pH 7.5, 100 mM NaCl, containing 0.5 mg/mL BSA and 20 × CMC individual detergents). A further 15 ml scintillation fluid was added and receptor-bound [^3^H]-DHA was measured with a scintillation counter (Beckman). The [^3^H]-DHA binding capacity of the receptor was expressed as a column graph. The experiment was carried out in triplicate.

### Determination of MelB stability and functionality

The *E. coli* DW2 strain (Δ*melB* and Δ*lacZY*) harboring pK95ΔAHB/WT MelB_St_/CH_10_, encoding the wild-type melibiose permease of *Salmonella typhimurium* (MelB_St_) carrying a C-terminal 10-His tag was used for this study^43, 53^. Membranes containing MelB_St_ (~10 mg/mL) in a buffer (20 mM sodium phosphate, pH 7.5, 200 mM NaCl, 10% glycerol and 20 mM melibiose) were treated with individual detergents [DDM, TMG-As (TMG-A11, TMG-A12, TMG-A13 and TMG-A14), or TMG-Ts (TMG-T11, TMG-T12, TMG-T13 and TMG-T14)] at 1.5% (w/v). The samples were then incubated at four different temperatures (0, 45, 55, and 65 °C) for 90 min, followed by ultracentrifugation at 355,590 g in a Beckman OptimaTM MAX ultracentrifuge using a TLA-100 rotor for 45 min at 4 °C. An equal amount of total membrane proteins (20 μg) was analysed on an SDS-15% PAGE gel. MelB_St_ was detected by immunoblotting with a Penta-His-HRP antibody (Qiagen, Germantown, MD). For the Trp → D^2^G FRET assay, the right-side-out (RSO) membrane vesicles were prepared from *E. coli* DW2 cells containing MelB_St_ or MelB_Ec_ by osmotic lysis^43, 54^. D^2^G (2′-(*N*-dansyl)aminoalkyl-1-thio-β-d-galactopyranoside) was provided by Drs. Gerard Leblanc and H. Ronald Kaback. RSO membrane vesicles in buffer (pH 7.5) containing 100 mM KP_i_ and 100 mM NaCl at a protein concentration of 1 mg/ml were treated with 1.0% DDM, TMG-A12, or TMG-A13 at 23 °C for 30 min and subjected to ultracentrifugation using a TLA 120.2 rotor at >300,000 g for 45 min at 4 °C. The supernatants were directly used for the Trp → D^2^G FRET experiments using an Amico-Bowman Series 2 (AB2) Spectrofluorometer. Trp residues were excited at 290 nm and emission was recorded at 465 nm and 490 nm for MelB_Ec_ and MelB_St_, respectively. On a time trace, 10 μM D^2^G was added at 1-min time point, and excess amount of melibiose or equal volume of water were added at 2-min time point.

## Electronic supplementary material

Supplementary information
