# Supplementary material for: Tandem malonate-based glucosides (TMGs) for membrane protein structural studies
Source: Sci Rep. 2017 Jun 21;7:3963. doi: 10.1038/s41598-017-03809-3 (PMC5479785; doi:10.1038/s41598-017-03809-3)

Supplementary information

Tandem malonate-based glucosides (TMGs) for membrane protein structural studies

Hazrat Hussain, Jonas S. Mortensen, Yang Du, Claudia Santillan, Orquidea Ribeiro, Juyeon Go, Parameswaran Hariharan, Claus J. Loland, Lan Guan, Brian K. Kobilka, Bernadette Byrne, and Pil Seok Chae*

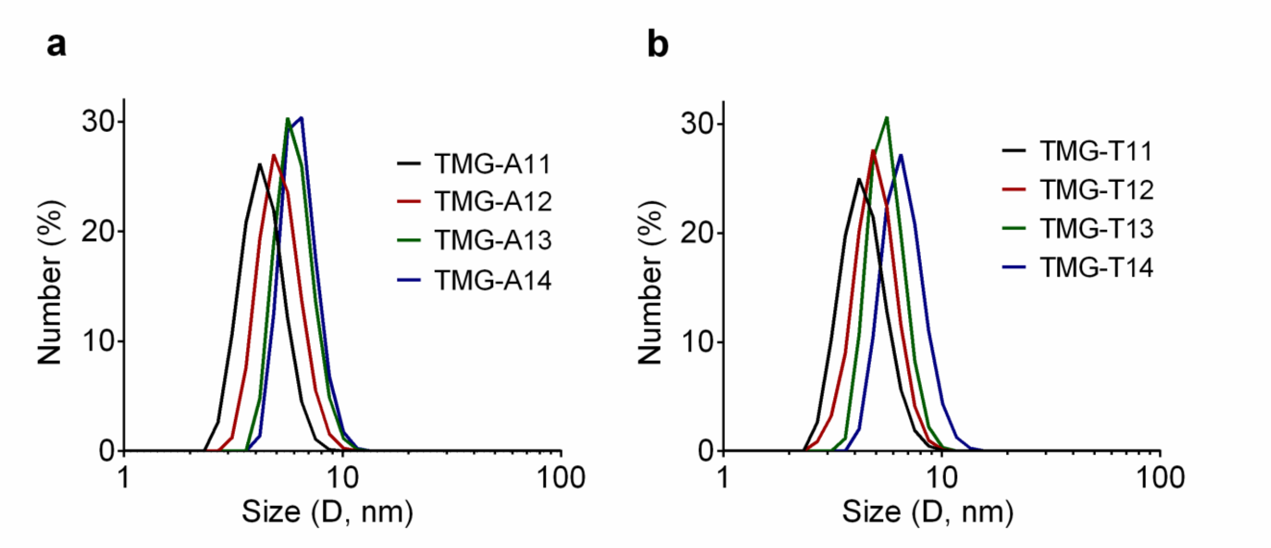

Supplementary Figure S1. Number-averaged dynamic light scattering profiles of micelles formed by TMG-As (a) and TMG-Ts (b). Each TMG-A (TMG-A11, TMG-A12, TMG-A13 or TMG-A14) or TMG-T (TMG-T11, TMG-T12, TMG-T13 or TMG-T14) was used at 1.0 wt%. Time-dependent fluctuation in the scattered light intensity was analyzed by autocorrelation, giving the translational diffusion coefficient (*D*). The hydrodynamic radii (R_h) of detergent micelles were correlated with the diffusion constant *via* the Stokes-Einstein equation.

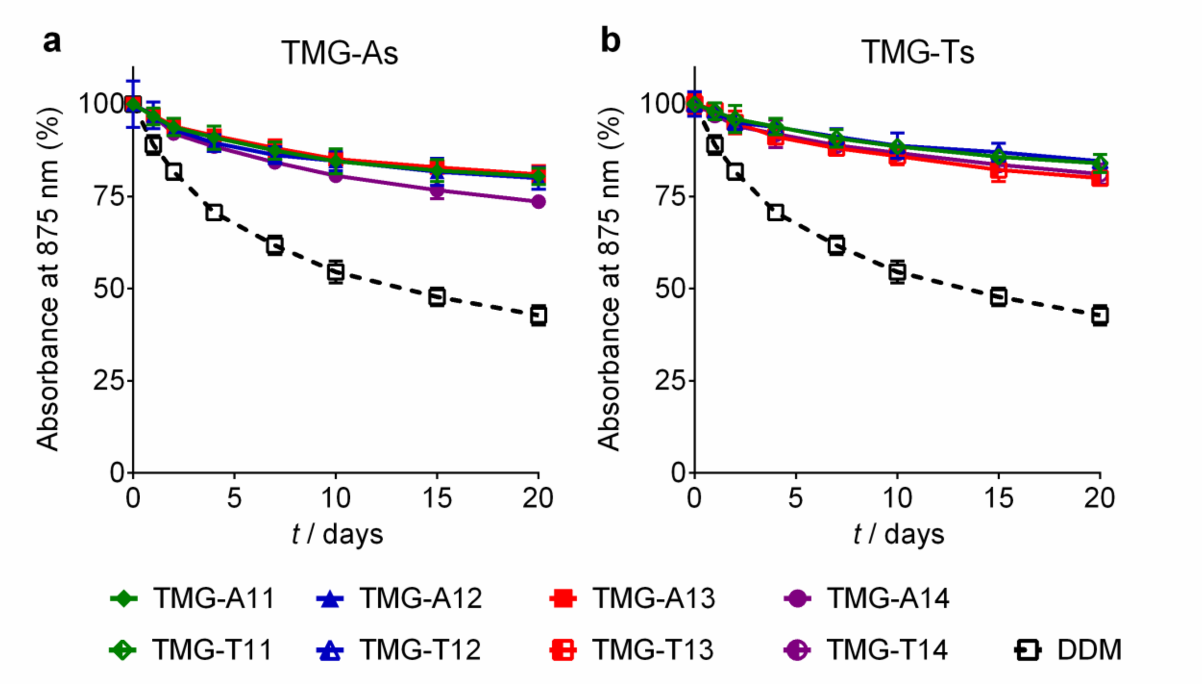

Supplementary Figure S2. Long-term stability of the *R. capsulatus* superassembly solubilized in individual TMG-As (TMG-A11, TMG-A12, TMG-A13 and TMG-A14) (a) and TMG-Ts (TMG-T11, TMG-T12, TMG-T13 and TMG-T14) (b). A conventional detergent (DDM) was used as a control. Protein stability was assessed *via* changes in absorbance value at 875 nm (A_{875}) over a 20-day incubation at 25℃. The results are expressed as % absorbance relative to the value on day 0 (mean ± SEM, n = 2).

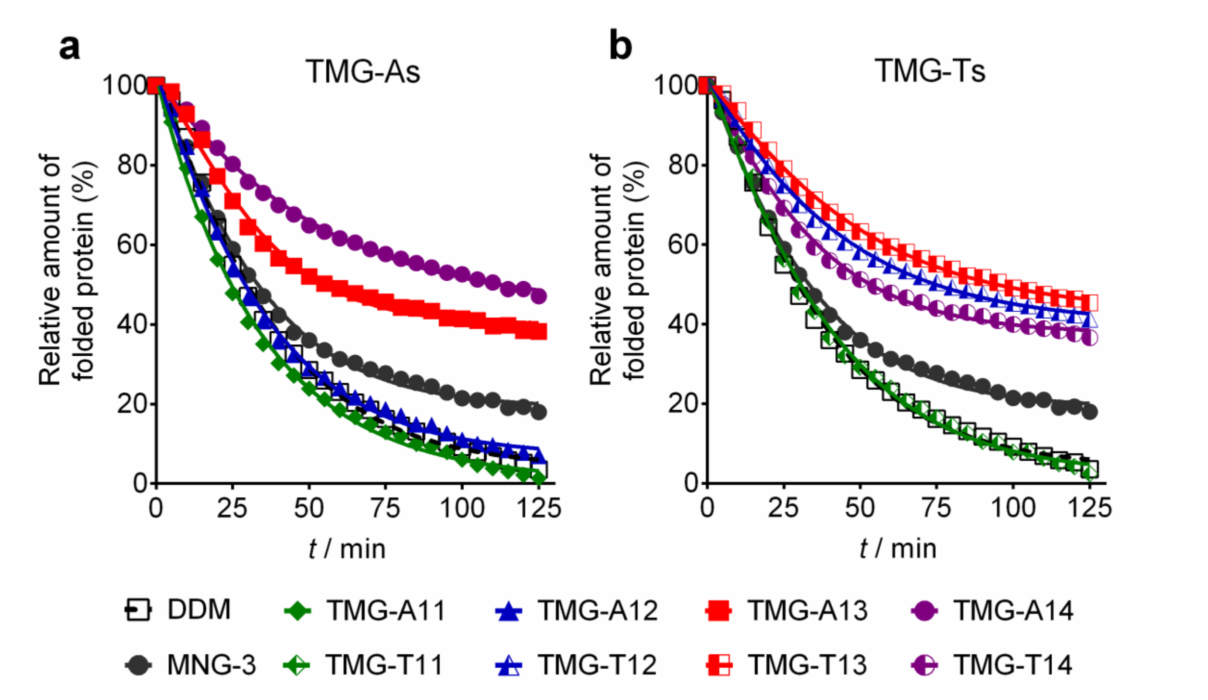

Supplementary Figure S3. Thermal denaturation profiles of UapA solubilized in individual novel agents (TMG-As (a) and TMG-Ts (b)), MNG-3, DDM used at CMC+0.2 wt%. The relative amounts of the folded protein were estimated by measuring solution fluorescence intensity *via* CPM assay during a 125-min incubation at 40°C. Mean standard deviation (n = 3) for DDM, TMG-A11, TMG-A12, TMG-A13, TMG-A14, MNG-3, TMG-T11, TMG-T12, TMG-T13 and TMG-T14 are 2.4, 4.5, 4.3, 3.7, 4.1, 2.7, 3.3, 7.7, 5.3, 5.6, respectively.

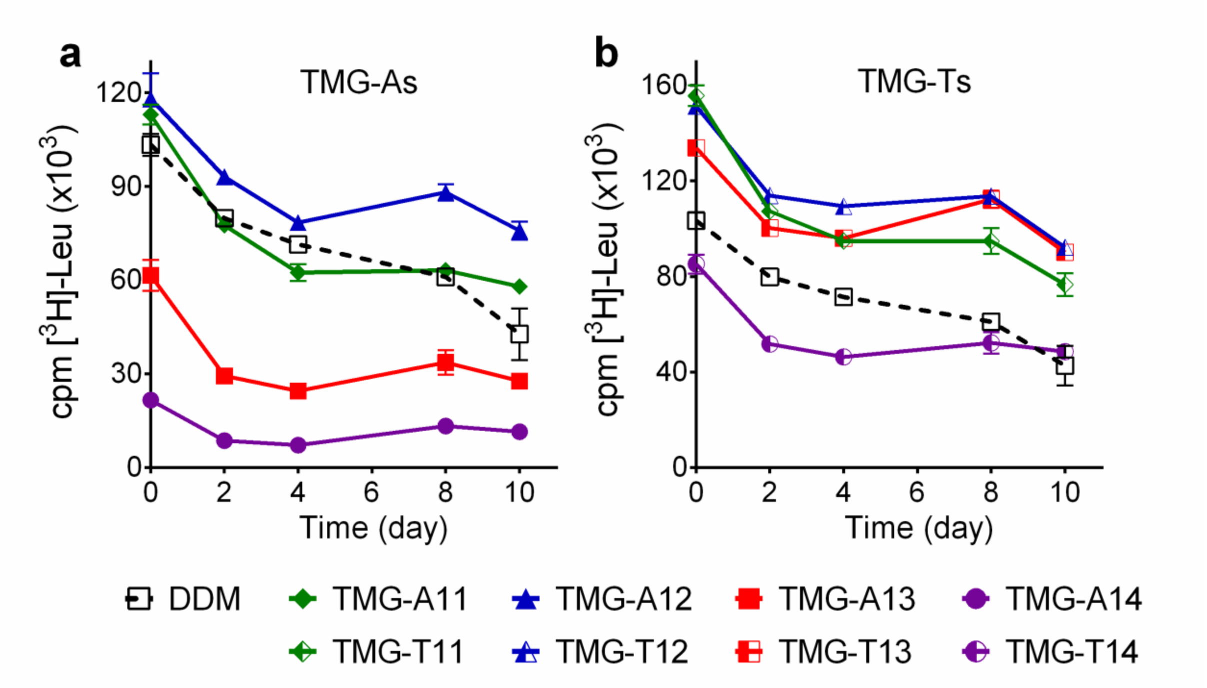

Supplementary Figure S4. Long-term stability of LeuT solubilized in DDM, individual TMG-As (TMG-A11, TMG-A12, TMG-A13 and TMG-A14), (a) or individual TMG-Ts (TMG-T11, TMG-T12, TMG-T13 and TMG-T14) (b). LeuT stability was measured based on the ability of the transporter to bind a radiolabelled substrate ([^{3}H]-Leu) *via* scintillation proximity assay (SPA). The transporter activity was measured in regular intervals over a 10-day incubation at room temperature. The results are expressed as specific binding of [^{3}H]-Leu (mean ± SEM, n = 2). All detergents were used at CMC+0.2 wt% for comparison.

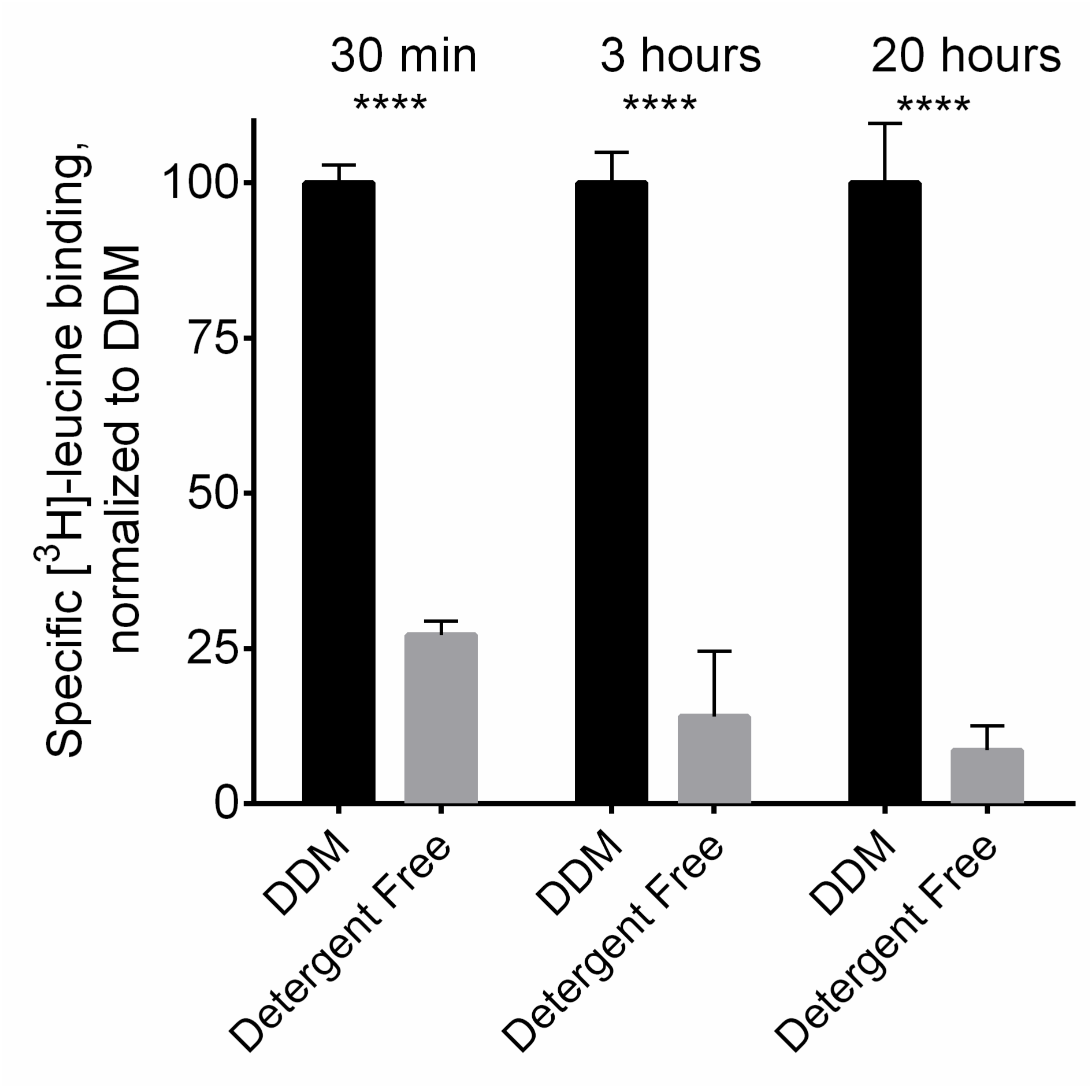

Supplementary Figure S5. Time course LeuT stability solubilized in DDM or detergent-free condition. LeuT stability was measured based on the ability of the transporter to bind a radiolabeled substrate ([^{3}H]-Leu) *via* scintillation proximity assay (SPA). Transporter activity was measured in regular intervals over a 20-hour incubation at room temperature. The data of detergent-free condition expressed as specific binding of [^{3}H]-Leu were normalized to those of DDM (mean ± SEM, n = 3) at the individual time points.

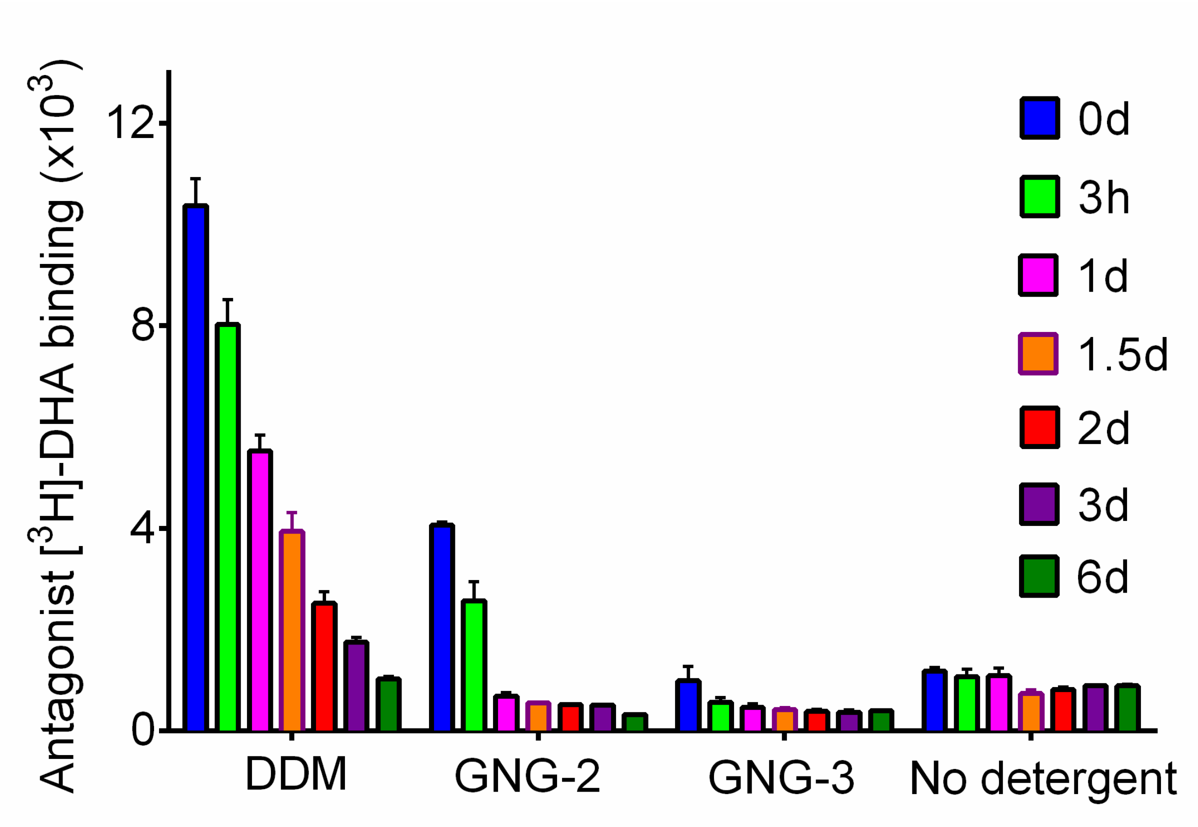

Supplementary Figure S6. Time course β_2AR stability solubilized in DDM, GNG-2, GNG-3 or detergent-free condition. β_2AR stability was monitored by measuring the ability of the receptor to bind the antagonist ([^{3}H]-DHA). Receptor activity was measured in regular intervals in the course of a 6-day incubation at room temperature. Error bars, SEM, *n* = 3.

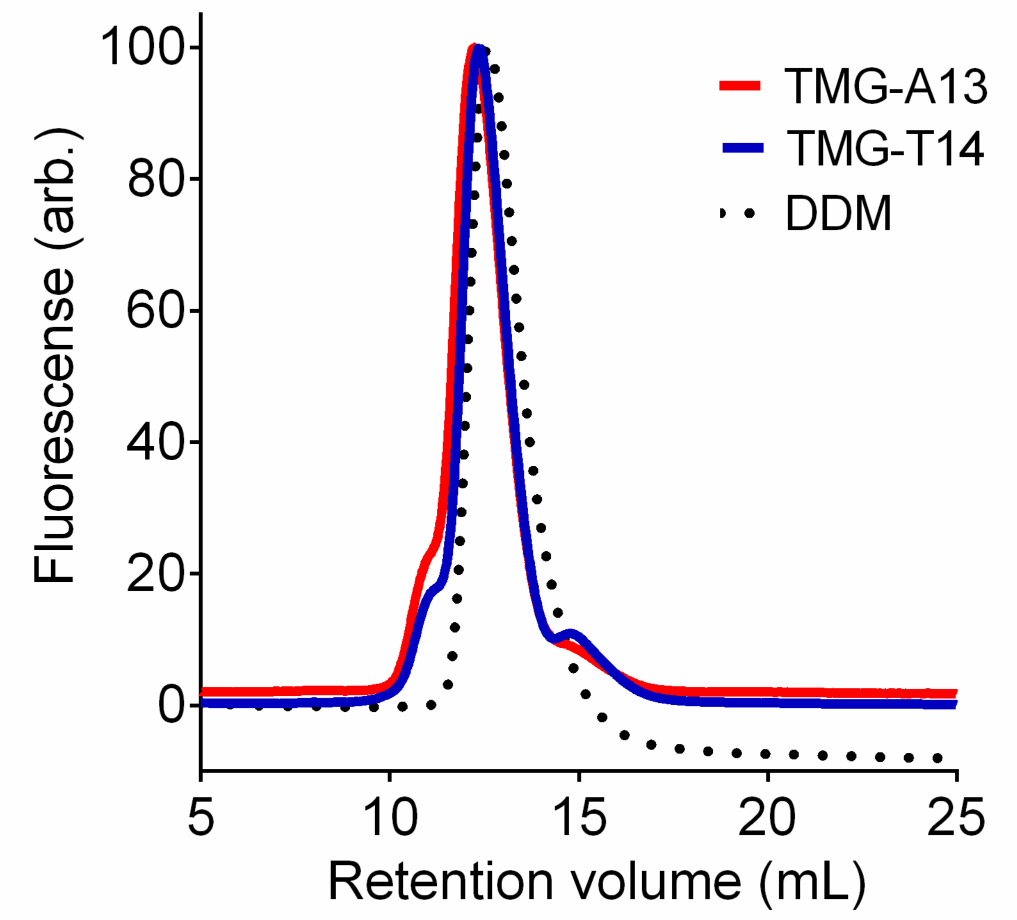

Supplementary Figure S7. Size exclusion chromatography (SEC) profiles of β_2AR in selected novel detergents (TMG-A13 and TMG-T14) and DDM. Detergent was exchanged from DDM to a TMG (TMG-A13 or TMG-T14) before applying each detergent-solubilized receptor for SEC column.

Table S1. Concentrations of target membrane proteins (μM) and residual DDM (wt%) after sample dilution into buffer solutions containing the individual novel agents (TMG-As and TMG-Ts).

Protein	Protein concentration (μM)	Residual DDM concentration (wt%)[a]
LHI-RC	0.2	0.005[a]
UapA	0.55	0.011[a]
LeuT	2.2	0.030[b]
β_2AR	0.002	0.0007[a]

[a] Value was calculated assuming that 400 DDMs are associated with a single protein. [b] Value was obtained using the 226 DDMs/protein, known for LeuT.[1]

Supplementary synthetic protocol

Supplementary Scheme 1

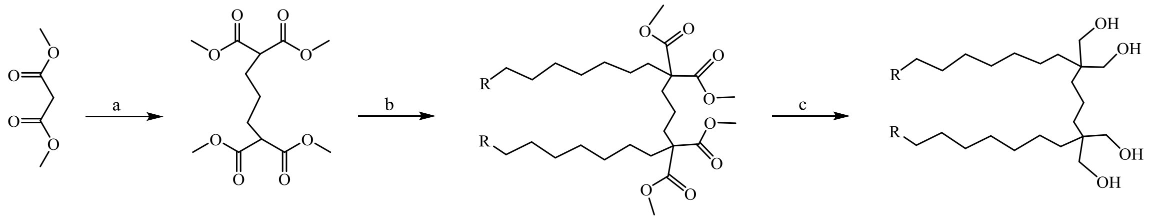

A

B1: R = *n*-C_4H_9
B2: R = *n*-C_5H_{11}
B3: R = *n*-C_6H_{13}
B4: R = *n*-C_7H_{15}

C1: R = *n*-C_4H_9
C2: R = *n*-C_5H_{11}
C3: R = *n*-C_6H_{13}
C4: R = *n*-C_7H_{15}

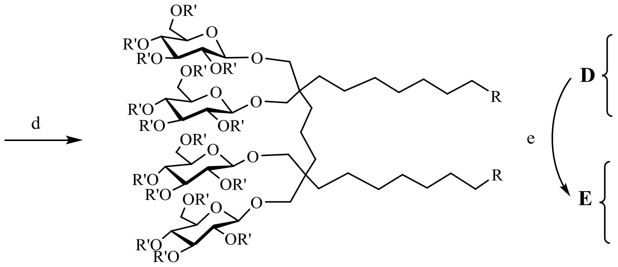

D
TMG-A11a: R = *n*-C_4H_9, R' = benzoyl
TMG-A12a: R = *n*-C_5H_{11}, R' = benzoyl
TMG-A13a: R = *n*-C_6H_{13}, R' = benzoyl
TMG-A14a: R = *n*-C_7H_{15}, R' = benzoyl

E
TMG-A11: R = *n*-C_4H_9, R' = H
TMG-A12: R = *n*-C_5H_{11}, R' = H
TMG-A13: R = *n*-C_6H_{13}, R' = H
TMG-A14: R = *n*-C_7H_{15}, R' = H

a) DMF, K_2CO_3, 1,3-diiodopropane, 100 ^oC; b) DMF, NaH, RI, 50 ^oC; c) THF, $LiAlH_4$, RT; d) DCM, 2,4,6-collidine, AgOTf, perbenzoylated glucosylbromide, 0 ^oC; e) NaOMe, MeOH/DCM, RT.

*General protocol for the synthesis of tetramethyl pentane-1,1,5,5-tetracarboxylate (**A**; step a)*[2]

This reaction was carried out according to the reported protocol with slight modifications. To a stirring solution of K_2CO_3 (2.34 g, 16.9 mmol, 2.5 equiv.) and 1,3-diiodopopane (6.76 mmol, 1 equiv.) in anhydrous DMF (20 mL) was added dimethyl malonate (16.9 mmol, 2.5 equiv.). After 24 h of stirring at room temperature, the reaction vessel was transferred to preheated oil bath at 100 ^oC and further stirred for an additional 4 h. After completion (monitored by TLC) of the reaction, the reaction mixture was diluted with ether (100 mL), washed with water (2 x 100 mL) and brine (100 mL), and dried with anhydrous Na_2SO_4. Removal of solvent followed by column chromatography (EtOAc/hexane) on silica gel gave colorless oil (**A**) which solidified was on standing.

*General protocol for the alkylation of tetramethyl pentane-1,1,5,5-tetracarboxylate (**B**; step b)*

Alkylation of the tetramethyl pentane-1,1,5,5-tetracarboxylate (**A**) was carried out according to the literature.[3] To a stirring suspension of NaH (15.8 mmol, 2.4 equiv.) in dry DMF (25 mL)

was added tetramethyl pentane-1,1,5,5-tetracarboxylate (6.57 mmol, 1 equiv.). After 15 min of stirring, 1-iodoalkane (15.8 mmol, 2.4 equiv.) was added and stirred at room temperature overnight, followed by 5 h stirring at 50 ^{o}C. After reaction completion (monitored by TLC), the reaction was quenched by adding ice-cold saturated NH_4Cl and extracted with diethyl ether (150 mL). The organic layer was washed with water (2 x 100 mL), brine (100 mL) and dried with anhydrous Na_2SO_4. After complete evaporation of the solvent, the residue was purified by silica gel column chromatography (EtOAc/hexane) providing dialkylated tetramethyl pentane-1,1,5,5-tetracarboxylate (**B**) as an oily liquid.

*General protocol for the reduction of alkylated tetramethyl pentane-1,1,5,5-tetracarboxylate (**C**; step c)*

To a stirring suspension of $LiAlH_4$ (18.72 mmol, 6 equiv) in anhydrous THF (20 mL) at 0 ^{o}C was added a solution of dialkylated tetramethyl pentane-1,1,5,5-tetracarboxylate (C; 3.12 mmol, 1 equiv.) in THF (15 mL) dropwise over 15 min. The mixture was stirred at room temperature for 6 h. After completion (monitored by TLC), the reaction was successively quenched with MeOH, water, a 1 N aqueous HCl solution at 0 °C. The organic layer was extracted with DCM (200 mL) and washed with water (2 x 150 mL), brine (100 mL) and dried with anhydrous Na_2SO_4. After evaporation of organic solvent, the reaction mixture was purified by silica gel column chromatography (EtOAc/hexane), providing dialkyl-containing tetraol (**C**), as a white crystalline solid.

General procedure for glycosycosylation reactions[4] *(**D**; step d)*

Glycosylation was carried out according to a literature,[4] with some modifications. Briefly to a stirring solution of an alcohol derivative (C), and 2,4,6-collidine (3.0 equiv.) in CH_2Cl_2 (15 mL) was added AgOTf (5.0 equiv.) at 0 ^{o}C and stirred for 10 min. To this mixture a solution of perbenzoylated glucosylbromide (5.0 equiv) in CH_2Cl_2 (5 mL) was added dropwise. The reaction was allowed to stir for 30 min at 0 ^{o}C. After completion of the reaction (as detected by TLC), pyridine was added to quench the reaction. The reaction mixture was diluted with CH_2Cl_2 (20 mL) before being filtered over celite. The filtrate was washed successively with a 1 M aqueous $Na_2S_2O_3$ solution (40 mL), a 0.1 M aqueous HCl solution (40 mL), and brine (3 x 40 mL). Then the organic layer was dried with anhydrous Na_2SO_4 and the solvent was removed by rotary evaporation. The residue was purified by silica gel column chromatography (EtOAc/hexane), providing the desired product (**D**) as a glassy solid.

De-O-benzoylation under Zemplén's conditions[4] *(**E**; step e)*

De-*O*-benzoylation reaction was carried out by following a literature protocol.[4] *O*-benzoylated compound (**D**) was dissolved in anhydrous CH_2Cl_2 and MeOH was added

dropwise untill appearance of persistent precipitation. To the reaction mixture was added the required amount of a methanolic solution of 0.5 M NaOMe such that the final concentration of NaOMe was 0.05 M. Methanolic solution was added in such a way that precipitation was avoided. The reaction mixture was allowed to stir for 6 h at room temperature. After completion of reaction (monitored by TLC) the reaction mixture was neutralized with amberlite IR-120 (H^+ form) resin. The resin was removed by filtration and washed with MeOH, and solvent was removed from the combined filtrate *in vacuo*. The residue was purified by recrystallization using CH_2Cl_2/MeOH/diethyl ether, affording fully de-*O*-benzoylated product (**E**) as a white solid.

Tetramethyl pentane-1,1,5,5-tetracarboxylate (A) was synthesized according to the general synthetic procedure for the synthesis of tetramethyl pentane-1,1,5,5-tetracarboxylate in 93% yield. **^{1}H NMR** (400 MHz, $CDCl_3$): δ 3.73 (s, 12H), 3.36 (t, *J* = 8.0 Hz, 2H), 1.96-1.90 (m, 4H), 1.39-1.33 (m, 2H); **^{13}C NMR** (100 MHz, $CDCl_3$): δ 170.0, 52.6, 51.4, 28.4, 25.1.

Tetramethyl heptacosane-12,12,16,16-tetracarboxylate (B1) was synthesized according to the general synthetic protocol for the alkylation of tetramethyl pentane-1,1,5,5-tetracarboxylate in 82% yield. **^{1}H NMR** (400 MHz, $CDCl_3$): δ 3.69 (s, 12H), 1.88-1.78 (m, 8H), 1.38-1.10 (m, 46H), 0.84 (t, *J* = 7.1 Hz, 6H); **^{13}C NMR** (100 MHz, $CDCl_3$): δ 172.4, 57.7, 52.5, 34.3, 33.0, 32.9, 32.7, 32.1, 30.0, 29.8, 29.6, 29.0, 28.4, 24.3, 22.9, 19.1, 14.3.

Tetramethyl nonacosane-13,13,17,17-tetracarboxylate (B2) was synthesized according to the general synthetic protocol for the alkylation of tetramethyl pentane-1,1,5,5-tetracarboxylate in 83% yield. **^{1}H NMR** (400 MHz, $CDCl_3$): δ 3.70 (s, 12H), 1.89-1.80 (m, 8H), 1.28-1.10 (m, 50H), 0.88 (t, *J* = 7.1 Hz, 6H); **^{13}C NMR** (100 MHz, $CDCl_3$): δ 172.3, 57.7, 52.5, 32.9, 32.7, 32.1, 30.7, 30.0, 29.9, 29.8, 29.6, 28.8, 24.3, 22.9, 19.1, 14.3.

Tetramethyl hentriacontane-14,14,18,18-tetracarboxylate (B3) was synthesized according to the general synthetic protocol for the alkylation of tetramethyl pentane-1,1,5,5-tetracarboxylate in 85% yield. **^{1}H NMR** (400 MHz, $CDCl_3$): δ 3.68 (s, 12H), 1.87-1.80 (m, 8H), 1.38-1.32 (m, 2H), 1.28 (s, 54H), 1.25-1.10 (m, 6H), 0.87 (t, *J* = 7.1 Hz, 6H); **^{13}C NMR** (100 MHz, $CDCl_3$): δ 172.4, 57.6, 52.5, 34.3, 33.1, 32.9, 32.7, 32.1, 30.0, 29.9, 29.7, 29.6, 29.0, 28.4, 24.3, 22.9, 19.1, 14.3.

Tetramethyl tritriacontane-15,15,19,19-tetracarboxylate (B4) was synthesized according to the general synthetic protocol for the alkylation of tetramethyl pentane-1,1,5,5-tetracarboxylate in 87%. **^{1}H NMR** (400 MHz, $CDCl_3$): δ 3.69 (s, 12H), 1.89-1.80 (m, 8H), 1.26-1.05 (m, 58H), 0.87 (t, *J* = 7.1 Hz, 6H); **^{13}C NMR** (100 MHz, $CDCl_3$): δ 172.4, 57.7, 52.5, 32.9, 32.8, 32.1, 30.0, 29.9, 29.8, 29.6, 24.3, 22.9, 19.1, 14.3.

2,6-bis(hydroxymethyl)-2,6-diundecylheptane-1,7-diol (C1) was synthesized according to the general synthetic procedure for the reduction of alkylated tetramethyl pentane-1,1,5,5-tetracarboxylate in 83% yield. **^{1}H NMR** (400 MHz, $CDCl_3$): δ 3.54 (s, 8H), 1.68-1.25 (m, 46H), 0.88 (t, J = 6.8 Hz, 6H); **^{13}C NMR** (100 MHz, $CDCl_3$): δ 32.1, 31.8, 30.8, 30.0, 29.8, 29.6, 27.0, 22.9, 19.1, 14.3.

2,6-didodecyl-2,6-bis(hydroxymethyl)heptane-1,7-diol (C2) was synthesized according to the general synthetic procedure for the reduction of alkylated tetramethyl pentane-1,1,5,5-tetracarboxylate in 83% yield. **^{1}H NMR** (400 MHz, $CDCl_3$): δ 3.49 (s, 8H), 1.66-1.24 (m, 50H), 0.88 (t, J = 6.8 Hz, 6H); **^{13}C NMR** (100 MHz, $CDCl_3$): δ 32.2, 31.9, 30.8, 30.0, 29.9, 29.6, 27.2, 23.0, 22.9, 14.4.

2,6-bis(hydroxymethyl)-2,6-ditridecylheptane-1,7-diol (C3) was synthesized according to the general synthetic procedure for the reduction of alkylated tetramethyl pentane-1,1,5,5-tetracarboxylate in 85% yield. **^{1}H NMR** (400 MHz, $CDCl_3$): δ 3.49 (s, 8H), 1.67-1.10 (m, 54H), 0.88 (t, J = 6.8 Hz, 6H); **^{13}C NMR** (100 MHz, $CDCl_3$): δ 32.2, 31.8, 30.9, 30.1, 29.9, 29.6, 27.0, 26.6, 22.9, 14.3.

2,6-bis(hydroxymethyl)-2,6-ditetradecylheptane-1,7-diol (C4) was synthesized according to the general synthetic procedure for the reduction of alkylated tetramethyl pentane-1,1,5,5-tetracarboxylate in 86% yield. **^{1}H NMR** (400 MHz, $CDCl_3$): δ 3.55 (s, 8H), 1.67-1.16 (m, 58H), 0.88 (t, J = 6.8 Hz, 6H); **^{13}C NMR** (100 MHz, $CDCl_3$): δ 32.2, 31.9, 31.1, 30.1, 29.9, 29.6, 27.1, 26.7, 22.9, 14.3.

TMG-A11a was synthesized according to the general synthetic procedure for the glycosylation reaction in 54% yield. **^{1}H NMR** (400 MHz, $CDCl_3$): δ 8.23-8.19 (m, 2H), 8.10-8.02 (m, 4H), 8.01-7.85 (m, 16H), 7.84-7.76 (m, 8H), 7.53-7.39 (m, 10H), 7.38-7.28 (m, 10H), 7.27-7.18 (m, 16H), 7.17-7.10 (m, 8H), 7.09-7.05 (m, 6H), 5.72-5.57 (m, 6H), 5.56-5.52 (m, 2H), 4.62-4.42 (m, 6H), 3.81-3.76 (m, 2H), 3.49-3.43 (m, 2H), 3.19-3.01 (m, 2H), 2.96-2.93 (m, 2H), 1.45-0.91 (m, 46H), 0.88 (t, J = 6.7 Hz, 6H); **^{13}C NMR** (100 MHz, $CDCl_3$): δ 166.1, 166.0, 165.9, 165.8, 165.2, 164.8, 164.7, 133.7, 133.5, 133.3, 133.0, 130.2, 130.1, 129.9, 129.8, 129.7, 129.4, 129.1, 129.0, 128.8, 128.7, 128.6, 128.5, 128.4, 128.1, 128.0, 101.9, 101.6, 101.5, 72.9, 72.7, 72.5, 72.0, 71.7, 71.3, 70.0, 69.7, 69.5, 69.3, 62.9, 60.5, 40.6, 32.1, 31.0, 30.9, 30.7, 30.4, 30.0, 29.6, 22.8, 22.5, 14.3.

TMG-A12a was synthesized according to the general synthetic procedure for the glycosylation reaction in 53% yield. **^{1}H NMR** (400 MHz, $CDCl_3$): δ 8.18-8.16 (m, 2H), 8.02-7.94 (m, 4H), 7.92-7.72 (m, 16H), 7.71-7.56 (m, 8H), 7.54-7.48 (m, 10H), 7.43-7.37 (m, 10H), 7.29-7.24 (m, 16H), 7.21-7.10 (m, 8H), 5.66-5.41 (m, 8H), 4.48-4.34 (m, 6H), 3.79-

3.74 (m, 2H), 3.51-3.45 (m, 2H), 2.94-2.90 (m, 2H), 1.27-1.15 (m, 50H), 0.86 (t, *J* = 7.1 Hz, 6H); **^{13}C NMR** (100 MHz, $CDCl_3$): δ 166.0, 165.9, 165.8, 165.7, 165.4, 165.2, 164.8, 164.7, 164.2, 133.7, 133.5, 133.2, 133.0, 130.4, 130.2, 130.0, 129.8, 129.7, 129.6, 129.4, 129.1, 129.0, 128.8, 128.6, 128.5, 128.4, 128.3, 128.1, 128.0, 101.6, 101.5, 72.8, 72.6, 72.5, 72.0, 71.7, 71.4, 70.0, 69.7, 69.5, 69.3, 62.3, 40.6, 32.0, 30.9, 30.7, 30.4, 30.0, 29.9, 29.5, 22.8, 22.5, 14.3.

TMG-A13a was synthesized according to the general synthetic procedure for the glycosylation reaction in 53% yield. **^{1}H NMR** (400 MHz, $CDCl_3$): δ 8.23-8.19 (m, 2H), 8.01-7.95 (m, 4H), 7.93-7.85 (m, 16H), 7.73-7.70 (m, 8H), 7.54-7.48 (m, 6H), 7.47-7.42 (m, 10H), 7.41-7.35 (m, 16H), 7.34-7.23 (m, 8H), 7.21-7.17 (m, 6H), 5.65-5.59 (m, 8H), 5.52-5.24 (m, 2H), 4.46-4.33 (m, 6H), 3.81-3.73 (m, 2H), 3.51-3.46 (m, 2H), 3.10-3.07 (m, 2H), 2.93-2.88 (m, 2H), 1.48-0.93 (m, 54H), 0.86 (t, *J* = 6.9 Hz, 6H); **^{13}C NMR** (100 MHz, $CDCl_3$): δ 166.1, 166.0, 165.9, 165.2, 165.1, 164.8, 164.7, 133.7, 133.5, 133.3, 133.2, 130.2, 130.0, 129.8, 129.7, 129.5, 129.3, 129.1, 129.0, 128.7, 128.6, 128.5, 128.4, 128.3, 128.1, 101.6, 101.5, 72.6, 72.0, 71.6, 71.4, 70.0, 69.7, 63.3, 62.9, 60.4, 53.6, 40.6, 32.0, 30.9, 30.7, 30.4, 30.0, 29.9, 29.8, 29.6, 22.8, 22.5, 14.3.

TMG-A14a was synthesized according to the general synthetic procedure for the glycosylation reaction in 52% yield. **^{1}H NMR** (400 MHz, $CDCl_3$): δ 8.24-8.20 (m, 2H), 8.02-7.96 (m, 4H), 7.95-7.89 (m, 16H), 7.75-7.71 (m, 8H), 7.54-7.48 (m, 6H), 7.47-7.41 (m, 10H), 7.39-7.35 (m, 16H), 7.34-7.23 (m, 8H), 7.21-7.17 (m, 6H), 5.65-5.60 (m, 8H), 5.54-5.43 (m, 2H), 4.46-4.33 (m, 6H), 3.82-3.73 (m, 2H), 3.53-3.47 (m, 2H), 3.14-3.09 (m, 2H), 2.95-2.86 (m, 2H), 1.49-0.94 (m, 58H), 0.86 (t, *J* = 7.1 Hz, 6H); **^{13}C NMR** (100 MHz, $CDCl_3$): δ 166.1, 166.0, 165.9, 165.2, 165.1, 164.8, 164.7, 133.7, 133.5, 133.3, 133.2, 130.2, 130.0, 129.8, 129.7, 129.5, 129.3, 129.1, 129.0, 128.7, 128.6, 128.5, 128.4, 128.3, 128.1, 101.6, 101.5, 72.6, 72.0, 71.6, 71.4, 70.0, 69.7, 69.2, 63.3, 63.0, 62.6, 60.4, 53.6, 40.6, 32.0, 30.9, 30.7, 30.4, 30.0, 29.9, 29.8, 29.5, 22.8, 22.5, 21.1, 14.9, 14.3.

TMG-A11 was synthesized according to the general synthetic procedure for De-*O*-benzoylation under Zemplén's condition in 95% yield. **^{1}H NMR** (400 MHz, CD_3OD): δ 4.34 (d, *J* = 4.0 Hz, 4H), 3.92-3.85 (m, 4H), 3.75-3.63 (m, 8H), 3.48-3.35 (m, 4H), 3.25-3.19 (m, 4H), 1.33-1.22 (m, 46H), 0.88 (t, *J* = 7.2 Hz, 6H); **^{13}C NMR** (100 MHz, CD_3OD): δ 105.0, 78.2, 77.8 , 75.3, 75.2, 73.3, 71.7, 62.9, 42.3, 33.2, 32.6, 32.0, 31.9, 31.2, 31.1, 31.0, 30.7, 23.9, 14.6; **HRMS (EI):** calcd. for $C_{55}H_{104}O_{24}[M+Na]^+$ 1149.4130, found 1149.9616.

TMG-A12 was synthesized according to the general synthetic procedure for De-*O*-benzoylation under Zemplén's condition in 95% yield. **^{1}H NMR** (400 MHz, CD_3OD): δ 4.32

(d, J = 4.0 Hz, 4H), 3.88-3.83 (m, 4H), 3.75-3.65 (m, 8H), 3.48-3.35 (m, 4H), 3.24-3.17 (m, 4H), 1.39-1.15 (m, 50H), 0.88 (t, J = 6.6 Hz, 6H); **^{13}C NMR** (100 MHz, CD_3OD): δ 105.1, 78.3, 78.1, 77.9, 75.4, 75.2, 73.3, 71.9, 62.9, 42.3, 33.3, 32.7, 32.1, 31.2, 31.1, 31.0, 30.7, 24.0, 16.9, 14.7; **HRMS (EI):** calcd. for $C_{57}H_{108}O_{24}[M+Na]^+$ 1177.4670, found 1177.7233.

TMG-A13 was synthesized according to the general synthetic procedure for De-*O*-benzoylation under Zemplén's condition in 96% yield. **^{1}H NMR** (400 MHz, CD_3OD): δ 4.32 (d, J = 4.0 Hz, 4H), 3.88-3.82 (m, 4H), 3.75-3.63 (m, 8H), 3.48-3.35 (m, 4H), 3.22-3.18 (m, 4H), 1.34-1.12 (m, 54H), 0.90 (t, J = 8.0 Hz, 6H); **^{13}C NMR** (100 MHz, CD_3OD): δ 105.1, 78.3, 77.9, 75.4, 75.2, 73.4, 71.8, 62.9, 42.4, 33.3, 32.7, 32.1, 32.0, 31.2, 31.1, 31.0, 24.0, 14.7; **HRMS (EI):** calcd. for $C_{59}H_{112}O_{24}[M+Na]^+$ 1205.5210, found 1205.754.

TMG-A14 was synthesized according to the general synthetic procedure for De-*O*-benzoylation under Zemplén's condition in 96% yield. **^{1}H NMR** (400 MHz, CD_3OD): δ 4.34 (d, J = 4.0 Hz, 4H), 3.88-3.84 (m, 4H), 3.75-3.65 (m, 8H), 3.48-3.35 (m, 4H), 3.22-3.18 (m, 4H), 1.31-1.12 (m, 58H), 0.90 (t, J = 8.0 Hz, 6H); **^{13}C NMR** (100 MHz, CD_3OD): δ 105.1, 78.2, 77.8, 75.3, 75.2, 73.3, 71.8, 62.9, 42.4, 33.2, 32.6, 32.1, 31.9, 31.2, 31.1, 31.0, 30.7, 24.0, 14.7; **HRMS (EI):** calcd. for $C_{61}H_{116}O_{24}[M+Na]^+$ 1233.5750, found 1233.7858.

Supplementary Scheme 2

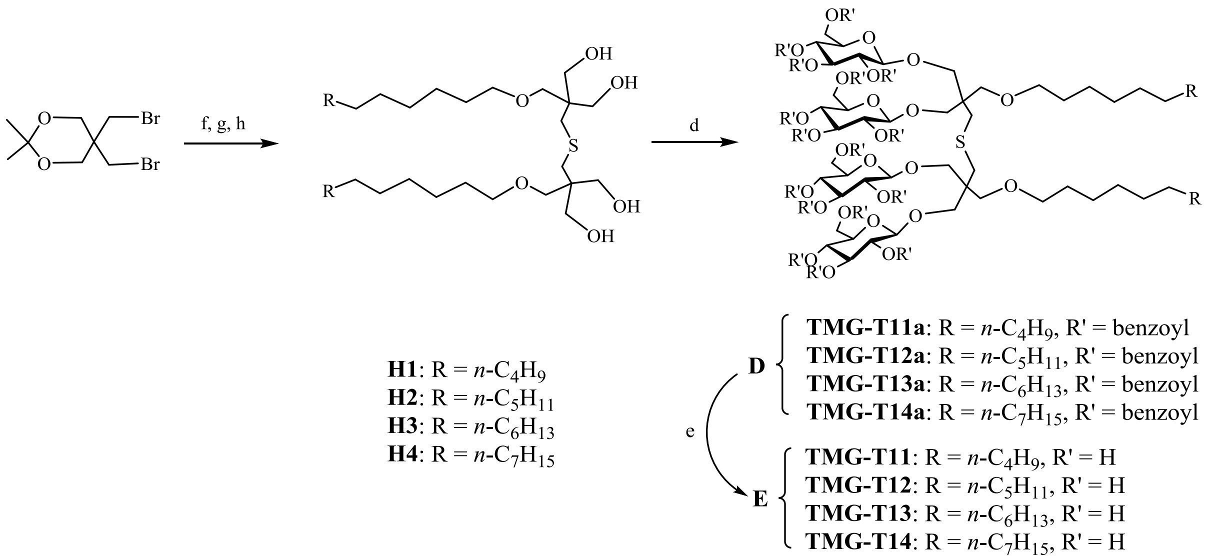

f) DMF, NaH, 1-iodoalkane, RT to 100 °C; g) DMF, KI, $Na_2S.9H_2O$, 90 °C; h) THF, $LiAlH_4$, RT; d) DCM, 2,4,6-collidine, AgOTf, perbenzoylated glucosylbromide, 0 °C; e) NaOMe, MeOH/DCM, RT.

General procedure for the synthesis of 2,2'-(thiobis(methylene))bis(2-(alkyloxy)methyl)propane-1,3-diol) (***H****; step f-h*)[5]

This compound was synthesized according to the literatures[5] with slight modification. To a solution of 1-alkanol (11.6 mmol, 1 equiv.) in DMF (25 mL) was added NaH (11.6 mmol, 1.2 equiv., 60%) at 0 °C. After stirring the mixture at room temperature for 30 min was 5,5-bis-bromomethyl-2,2-dimethyl-[1,3]dioxane (11.6 mmol, 1 equiv.) added. The reaction vessel was transferred to preheated oil bath at 100 °C and stirred further for 15 hr. After completion of the reaction (monitored by TLC), the reaction mixture was cooled to room temperature, quenched with ice-cold H_2O (50 mL) and extracted with ether (3 × 100 mL). The combined organic layer washed with brine (2 ×150 mL), dried with anhydrous Na_2SO_4 and then concentrated by rotary evaporation. The crude product (5.08 mmol, 1 equiv.) was dissolved in DMF (20 mL) and KI (5.08 mmol, 1 equiv.) was added to the solution. To this mixture was added $Na_2S.9H_2O$ (0.6 equiv.) in water (5 mL) followed by additional DMF (20 mL) and the mixture was stirred under N_2 at 90 °C for 20 hours. After cooling, the mixture was poured into water (300 mL) and extracted with ether (150 mL). The extract was washed sequentially with water (300 mL), 2.5% NaOH solution (300 mL), and brine (100 mL), dried with anhydrous Na_2SO_4. The reaction mixture was stirred with 3g silica gel, filtered and concentrated by rotary evaporation. To the residue dissolved in 1:1 mixture of CH_2Cl_2 and MeOH (50 mL) was added *p*-toluenesulfonic acid (*p*-TSA) monohydrate (200 mg) and left stirring at room temperature for 6 hr. After completion the reaction mixture was neutralized with $NaHCO_3$ solution, filtered and dried by rotary evaporation. Flash column chromatography (EtOAc/hexane) affords thioether-containing tetraol (**H**) as a white solid.

2,2'-(thiobis(methylene))bis(2-((undecyloxy)methyl)propane-1,3-diol) (H1) was synthesized according to the general synthetic procedure for the synthesis of 2,2'-(thiobis(methylene))bis(2-(alkyloxy)methyl)propane-1,3-diol) in 60% (three steps) yield. **^{1}H NMR** (400 MHz, $CDCl_3$): δ 3.65 (s, 4H), 3.51-3.31 (m, 8H), 3.30 (s, 8H), 1.58-1.49 (m, 4H), 1.29 (s, 32H), 0.89 (t, J = 6.8 Hz, 6H); **^{13}C NMR** (100 MHz, $CDCl_3$): δ 73.8, 73.3, 72.7, 66.1, 56.2, 49.8, 47.0, 45.4, 45.2, 35.6, 33.2, 30.9, 30.8, 30.6, 28.8, 27.5, 23.9, 21.5, 14.6.

2,2'-(thiobis(methylene))bis(2-((dodecyloxy)methyl)propane-1,3-diol) (H2) was synthesized according to the general synthetic procedure for the synthesis of 2,2'-(thiobis(methylene))bis(2-(alkyloxy)methyl)propane-1,3-diol) in 62% yield (three steps). **^{1}H NMR** (400 MHz, $CDCl_3$): δ 3.92 (s, 4H), 3.73-3.62 (m, 8H), 3.39 (s, 8H), 1.61-1.50 (m, 4H), 1.29 (s, 36H), 0.88 (t, J = 6.8 Hz, 6H); **^{13}C NMR** (100 MHz, $CDCl_3$): δ 73.9, 73.4, 72.2, 67.3, 64.9, 64.5, 48.2, 45.4, 45.1, 40.4, 35.6, 32.1, 29.8, 29.6, 29.5, 28.9, 26.3, 22.8, 14.3.

2,2'-(thiobis(methylene))bis(2-((tridecyloxy)methyl)propane-1,3-diol) (H3) was synthesized according to the general synthetic procedure for the synthesis of 2,2'-(thiobis(methylene))bis(2-(alkyloxy)methyl)propane-1,3-diol) in 63% yield (three steps). **^{1}H**

NMR (400 MHz, $CDCl_3$): δ 3.92 (s, 4H), 3.75-3.63 (m, 8H), 3.41 (s, 8H), 1.61-1.51 (m, 4H), 1.29 (s, 40H), 0.88 (t, J = 6.8 Hz, 6H); ^{13}**C NMR** (100 MHz, $CDCl_3$): δ 73.9, 73.3, 72.2, 67.5, 64.9, 64.6, 64.4, 48.2, 45.4, 45.1, 44.6, 40.7, 40.4, 35.6, 32.1, 29.8, 29.6, 29.5, 28.9, 26.2, 22.8, 17.3, 14.3.

2,2'-(thiobis(methylene))bis(2-((tetradecyloxy)methyl)propane-1,3-diol) (H4) was synthesized according to the general synthetic procedure for the synthesis of 2,2'-(thiobis(methylene))bis(2-(alkyloxy)methyl)propane-1,3-diol) in 65% yield (three steps). ^{1}**H NMR** (400 MHz, $CDCl_3$): δ 3.94 (s, 4H), 3.78-3.51 (m, 8H), 3.35 (s, 8H), 1.52-1.36 (m, 4H), 1.23 (s, 44H), 0.88 (t, J = 6.8 Hz, 6H); ^{13}**C NMR** (100 MHz, $CDCl_3$): δ 73.7, 73.2, 72.2, 71.8, 67.6, 67.1, 64.9, 64.7, 64.4, 48.3, 45.4, 45.1, 44.7, 40.8, 40.4, 38.8, 35.5, 32.0, 29.8, 29.6, 29.5, 28.8, 26.2, 22.8, 17.2, 14.3.

TMG-T11a was synthesized according to the general synthetic procedure for the glycosylation reaction in 52% yield. ^{1}**H NMR** (400 MHz, $CDCl_3$): δ 8.35-8.31 (m, 2H), 8.16-8.14 (m, 4H), 7.99-7.91 (m, 16H), 7.90-7.78 (m, 8H), 7.72-7.63 (m, 10H), 7.52-7.44 (m, 10H), 7.42-7.32 (m, 16H), 7.31-7.23 (m, 12H), 6.91-6.78 (m, 2H), 5.82-5.78 (m, 6H), 5.77-5.52 (m, 4H), 4.61-4.39 (m, 8H), 3.92-3.72 (m, 4H), 3.53-3.42 (m, 4H), 3.41-3.12 (m, 8H), 1.49-1.32 (m, 4H), 1.31-0.97 (m, 32H), 0.86 (t, J = 8.0 Hz, 6H); ^{13}**C NMR** (100 MHz, $CDCl_3$): δ 166.1, 166.0, 165.8, 165.7, 165.4, 165.2, 165.0, 164.9, 164.7, 133.8, 133.7, 133.4, 133.2, 133.1, 130.5, 130.3, 130.0, 129.8, 129.7, 129.6, 129.4, 129.3, 129.2, 129.1, 129.0, 128.7, 128.6, 128.5, 128.4, 128.0, 72.7, 72.6, 72.5, 72.1, 71.8, 71.6, 71.5, 70.0, 69.9, 60.5, 45.2, 32.1, 29.9, 29.8, 29.7, 29.6, 26.2, 22.8, 14.3.

TMG-T12a was synthesized according to the general synthetic procedure for the glycosylation reaction in 52% yield. ^{1}**H NMR** (400 MHz, $CDCl_3$): δ 8.37-8.31 (m, 2H), 8.19-8.11 (m, 4H), 8.01-7.91 (m, 16H), 7.81-7.76 (m, 8H), 7.74-7.62 (m, 12H), 7.51-7.42 (m, 10H), 7.41-7.34 (m, 16H), 7.33-7.19 (m, 12H), 6.91-6.78 (m, 2H), 5.79-5.65 (m, 6H), 5.64-5.46 (m, 4H), 4.59-4.42 (m, 8H), 3.89-3.71 (m, 4H), 3.43-3.22 (m, 4H), 3.21-3.12 (m, 8H), 1.49-1.32 (m, 4H), 1.31-0.97 (m, 36H), 0.86 (t, J = 6.4 Hz, 6H); ^{13}**C NMR** (100 MHz, $CDCl_3$): δ 165.4, 165.2, 165.0, 164.9, 133.8, 133.5, 133.3, 133.2, 133.1, 130.5, 130.3, 130.1, 129.9, 129.8, 129.7, 129.5, 129.3, 129.2, 128.8, 128.6, 128.5, 128.4, 128.1, 101.7, 101.1, 72.8, 72.6, 72.2, 71.9, 71.6, 71.5, 70.1, 69.9, 63.3, 45.2, 32.2, 30.0, 29.9, 29.8, 29.7, 29.6, 26.3, 22.9, 14.4.

TMG-T13a was synthesized according to the general synthetic procedure for the glycosylation reaction in 51% yield. ^{1}**H NMR** (400 MHz, $CDCl_3$): δ 8.37-8.31 (m, 2H), 8.21-8.12 (m, 4H), 8.01-7.91 (m, 16H), 7.83-7.76 (m, 8H), 7.72-7.62 (m, 12H), 7.50-7.42 (m, 10H), 7.41-7.35 (m, 16H), 7.34-7.20 (m, 12H), 6.83-6.76 (m, 2H), 5.81-5.64 (m, 6H), 5.63-

5.48 (m, 4H), 4.59-4.38 (m, 8H), 3.87-3.71 (m, 4H), 3.43-3.22 (m, 4H), 3.21-3.12 (m, 8H), 1.49-1.32 (m, 4H), 1.31-1.08 (m, 40H), 0.86 (t, J = 6.4 Hz, 6H); **^{13}C NMR** (100 MHz, $CDCl_3$): δ 166.1, 165.9, 165.7, 165.4, 165.2, 165.0, 164.9, 133.7, 133.4, 133.2, 133.1, 133.0, 130.5, 130.3, 130.0, 129.9, 129.8, 129.7, 129.6, 129.4, 129.3, 129.2, 129.1, 129.0, 128.8, 128.7, 128.6, 128.5, 128.4, 128.0, 72.8, 71.6, 71.5, 71.4, 60.5, 45.2, 32.1, 29.9, 29.8, 29.7, 29.5, 26.2, 22.8, 21.2, 14.3.

TMG-T14a was synthesized according to the general synthetic procedure for the glycosylation reaction in 51% yield. **^{1}H NMR** (400 MHz, $CDCl_3$): δ 8.37-8.32 (m, 2H), 8.21-8.14 (m, 4H), 8.01-7.89 (m, 16H), 7.83-7.78 (m, 8H), 7.73-7.64 (m, 12H), 7.52-7.40 (m, 10H), 7.39-7.32 (m, 16H), 7.31-7.18 (m, 12H), 6.83-6.76 (m, 2H), 5.81-5.63 (m, 6H), 5.62-5.49 (m, 4H), 4.57-4.34 (m, 8H), 3.88-3.72 (m, 4H), 3.45-3.23 (m, 4H), 3.22-3.12 (m, 8H), 1.50-1.32 (m, 4H), 1.31-1.04 (m, 44H), 0.86 (t, J = 6.8 Hz, 6H); **^{13}C NMR** (100 MHz, $CDCl_3$): δ 165.9, 165.8, 165.4, 165.2, 165.0, 164.9, 133.7, 133.5, 133.2, 133.1, 130.5, 130.3, 130.0, 129.9, 129.6, 129.4, 129.3, 129.2, 129.1, 129.0, 128.8, 128.6, 128.5, 128.4, 128.2, 128.0, 72.5, 72.3, 72.2, 71.8, 71.6, 71.5, 70.6, 69.9, 69.8, 69.2, 68.0, 63.9, 63.2, 45.2, 34.4, 32.1, 29.9, 29.8, 29.7, 29.6, 26.3, 22.9, 14.3.

TMG-T11 was synthesized according to the general synthetic procedure for De-*O*-benzoylation under Zemplén's condition in 94% yield. **^{1}H NMR** (400 MHz, CD_3OD): δ 4.37-4.31 (m, 4H), 3.95-3.83 (m, 8H), 3.78-3.62 (m, 4H), 3.61-3.51 (m, 4H), 3.49-3.35 (m, 12H), 3.31-3.19 (m, 4H), 1.61-1.49 (m, 4H), 1.41-1.20 (m, 32H), 0.90 (t, J = 7.2 Hz, 6H); **^{13}C NMR** (100 MHz, CD_3OD): δ 105.0, 104.8, 78.0, 77.8 , 75.2, 72.6, 71.6, 71.5, 71.4, 71.2, 70.7, 70.6, 63.0, 62.8, 46.4, 46.0, 33.2, 32.2, 31.0, 30.9, 30.8, 30.6, 27.5, 23.8, 14.6; **HRMS (EI):** calcd. for $C_{56}H_{106}O_{26}S[M+Na]^+$ 1227.4980 found 1227.6697.

TMG-T12 was synthesized according to the general synthetic procedure for De-*O*-benzoylation under Zemplén's condition in 95% yield. **^{1}H NMR** (400 MHz, CD_3OD): δ 4.38-31 (m, 4H), 3.97-3.84 (m, 8H), 3.74-3.68 (m, 4H), 3.65-3.53 (m, 4H), 3.47-3.37 (m, 12H), 3.29-3.17 (m, 4H), 1.61-1.49 (m, 4H), 1.41-1.20 (m, 36H), 0.90 (t, J = 7.2 Hz, 6H); **^{13}C NMR** (100 MHz, CD_3OD): δ 105.1, 105.0, 78.0, 77.8, 75.2, 72.7, 71.7, 71.6, 71.2, 70.8, 70.6, 70.4, 62.8, 46.4, 33.2, 31.0, 30.9, 30.8, 30.7, 27.6, 23.9, 14.7; **HRMS (EI):** calcd. for $C_{58}H_{110}O_{26}S[M+Na]^+$ 1255.5520 found 1255.7006.

TMG-T13 was synthesized according to the general synthetic procedure for De-*O*-benzoylation under Zemplén's condition in 96% yield. **^{1}H NMR** (400 MHz, CD_3OD): δ 4.37-4.28 (m, 4H), 3.98-3.84 (m, 8H), 3.73-3.62 (m, 4H), 3.62-3.51 (m, 4H), 3.47-3.38 (m, 12H), 3.27-3.17 (m, 4H), 1.62-1.49 (m, 4H), 1.41-1.20 (m, 40H), 0.90 (t, J = 7.2 Hz, 6H); **^{13}C**

NMR (100 MHz, CD_3OD): δ 106.0, 105.0, 104.9, 78.1, 78.0, 77.8 , 75.2, 74.7, 72.8, 72.7, 71.6, 71.5, 71.1, 62.8, 62.7, 46.4, 45.7, 33.2, 31.0, 30.9, 30.6, 27.5, 23.9, 14.6; **HRMS (EI):** calcd. for $C_{60}H_{114}O_{26}S[M+Na]^+$ 1283.7319 found 1283.7316.

TMG-T14 was synthesized according to the general synthetic procedure for De-*O*-benzoylation under Zemplén's condition in 96% yield. **^{1}H NMR** (400 MHz, CD_3OD): δ 4.38-4.28 (m, 4H), 3.97-3.82 (m, 8H), 3.74-3.65 (m, 4H), 3.64-3.51 (m, 4H), 3.48-3.38 (m, 12H), 3.29-3.16 (m, 4H), 1.62-1.50 (m, 4H), 1.42-1.18 (m, 44H), 0.90 (t, *J* = 7.2 Hz, 6H); **^{13}C NMR** (100 MHz, CD_3OD): δ 105.1, 105.0, 78.1, 77.8, 75.2, 72.7, 71.7, 71.6, 71.2, 70.8, 70.6, 70.4, 62.8, 46.4, 33.2, 31.0, 30.9, 30.8, 30.6, 27.6, 23.9, 14.7. **HRMS (EI):** calcd. for $C_{62}H_{118}O_{26}S[M+Na]^+$ 1311.6600 found 1311.7534.

References

1. Khelashvili, G., LeVine, M. V., Shi, L., Quick, M., Javitch, J. A. and Weinstein, H. *J. Am. Chem. Soc*. 2013, **135**, 14266-14275.
2. S. Muthusamy and B. Gnanaprakasam, *Tetrahed. Lett.*, 2005, **46**, 635-638.
3. M. Ehsan, Y. Du, N. J. Scull, E. Tikhonova, J. Tarrasch, J. S. Mortensen, C. J. Loland, G. Skiniotis, L. Guan, B. Byrne, B. K. Kobilka and P. S. Chae, *J. Am. Chem. Soc.*, 2016, **138**, 3789-3796.
4. P. S. Chae, S. G. F. Rasmussen, R. R. Rana, K. Gotfryd, R. Chandra, M. A. Goren, A. C. Kruse, S. Nurva, C. J. Loland, Y. Pierre, D. Drew, J.-L. Popot, D. Picot, B. G. Fox, L. Guan, U. Gether, B. Byrne, B. Kobilka and S. H. Gellman, *Nat. Methods*, 2010, **7**, 1003-1008.
5. M. T. Morgan, P. Bagchi and C. J. Fahrni, *J. Am. Chem. Soc.*, 2011, **133**, 15906-15909.

^{1}H NMR spectra of TMG amphipihles

TMG-A11

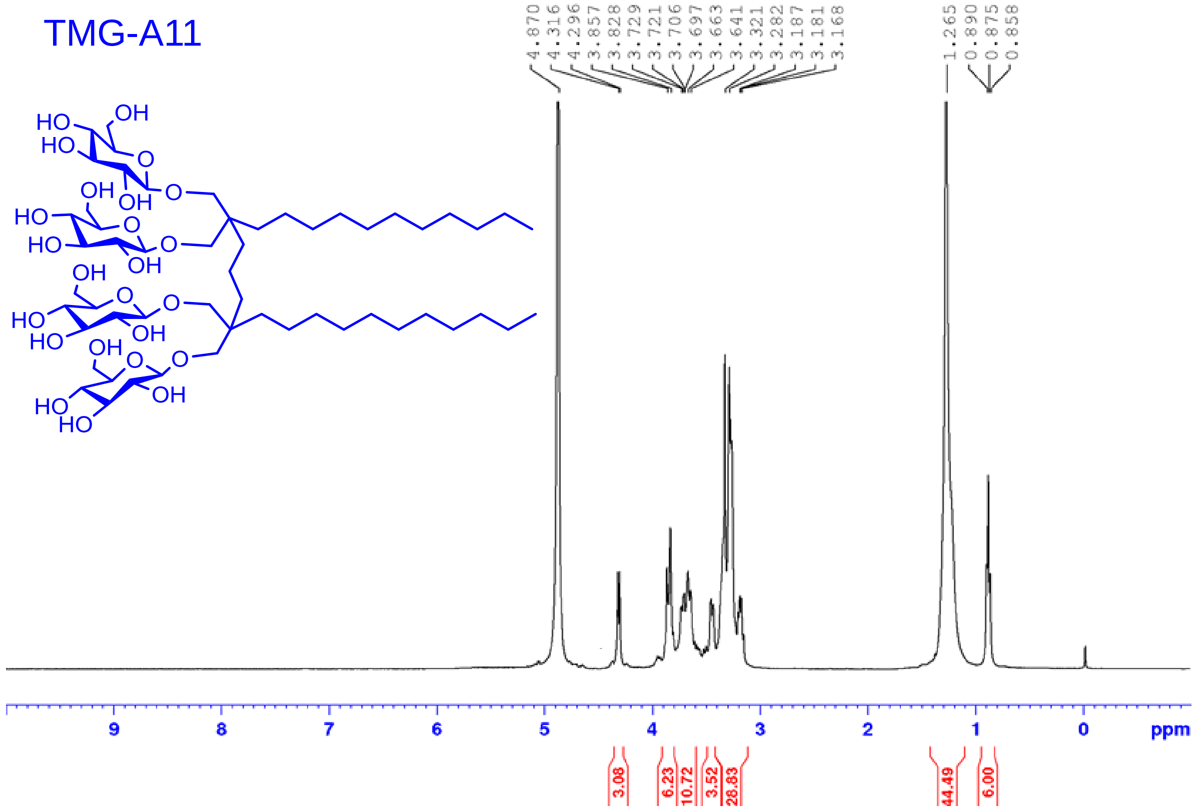

TMG-A12

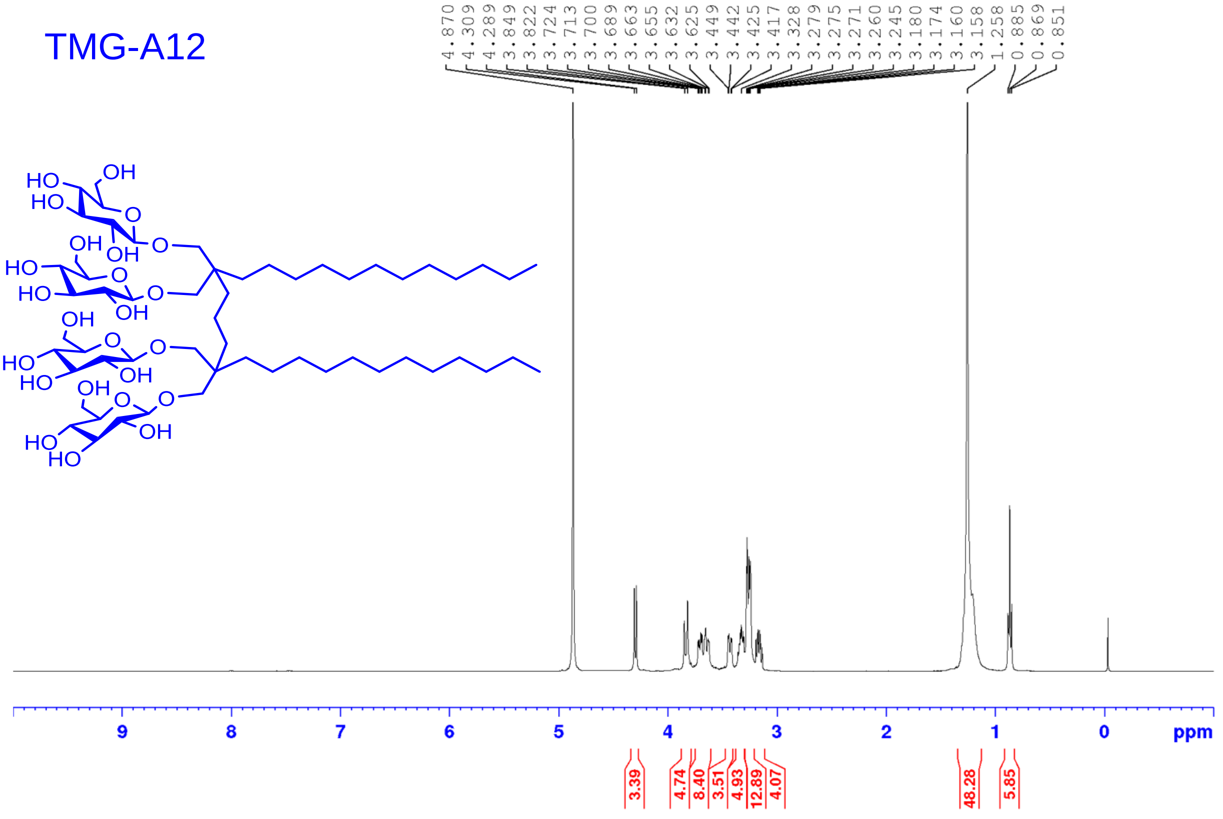

TMG-A13

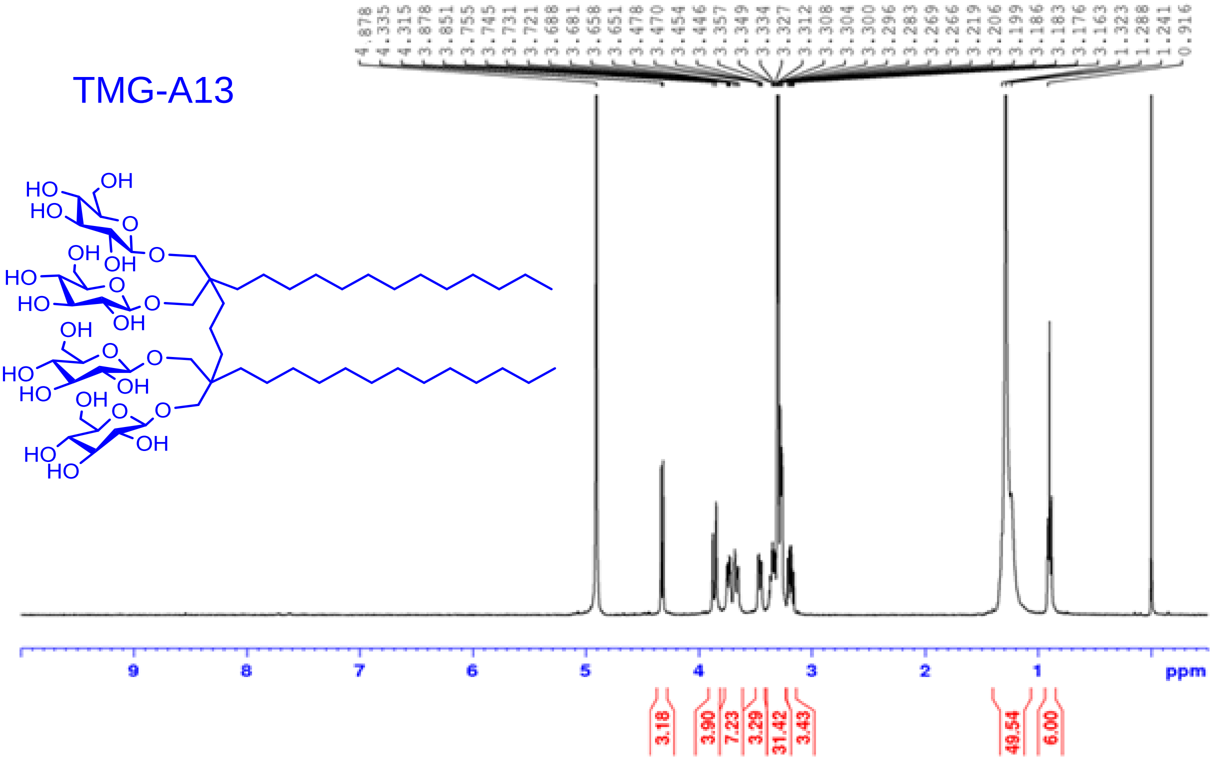

TMG-A14

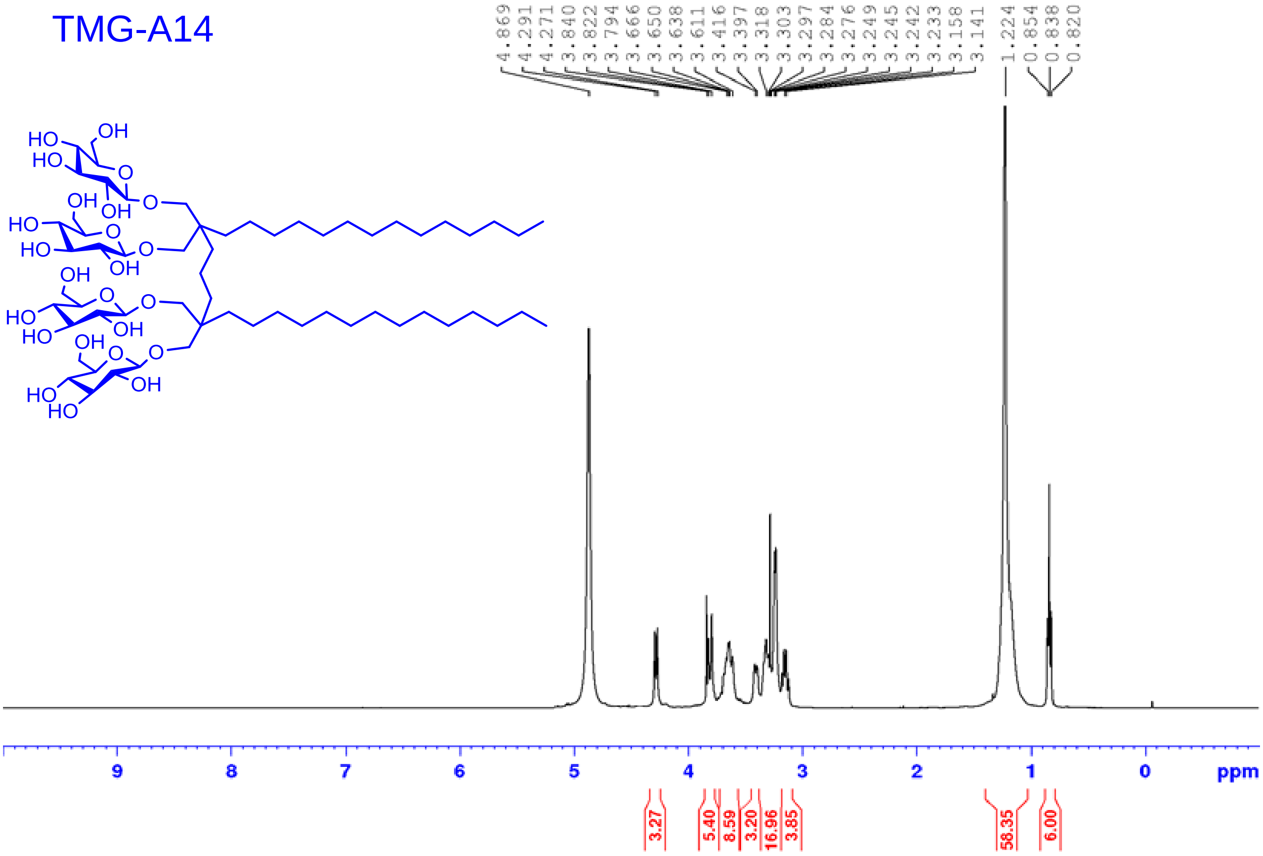

TMG-T11

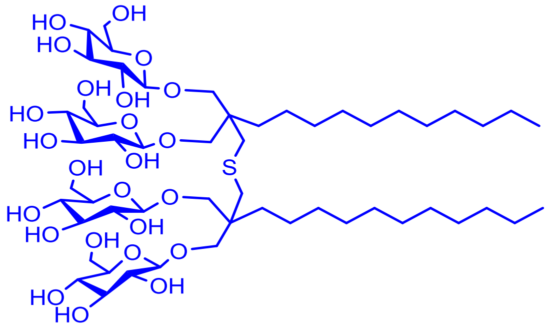

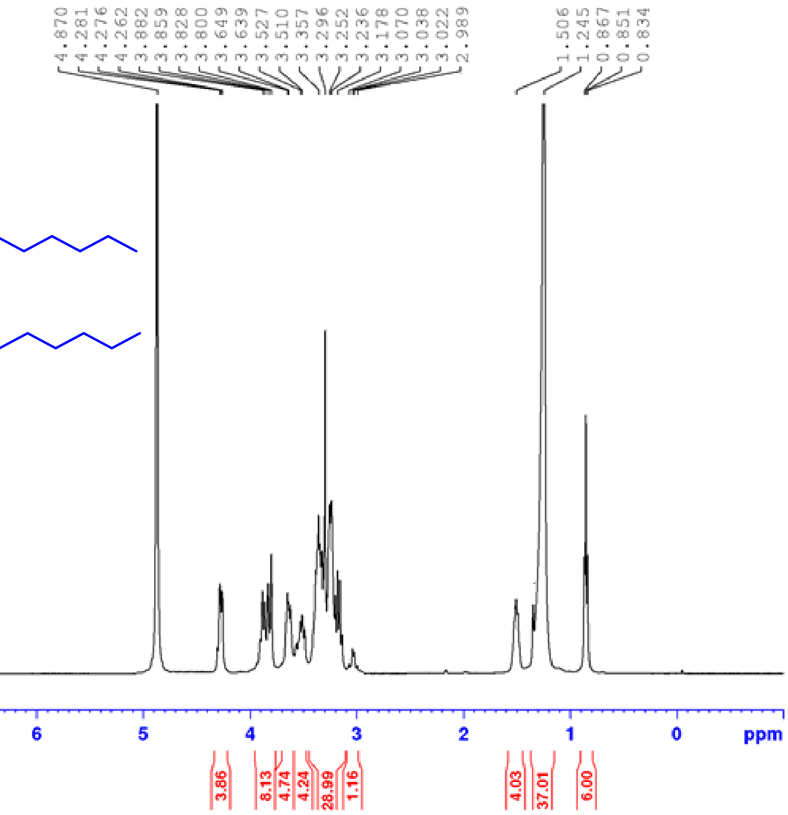

TMG-T12

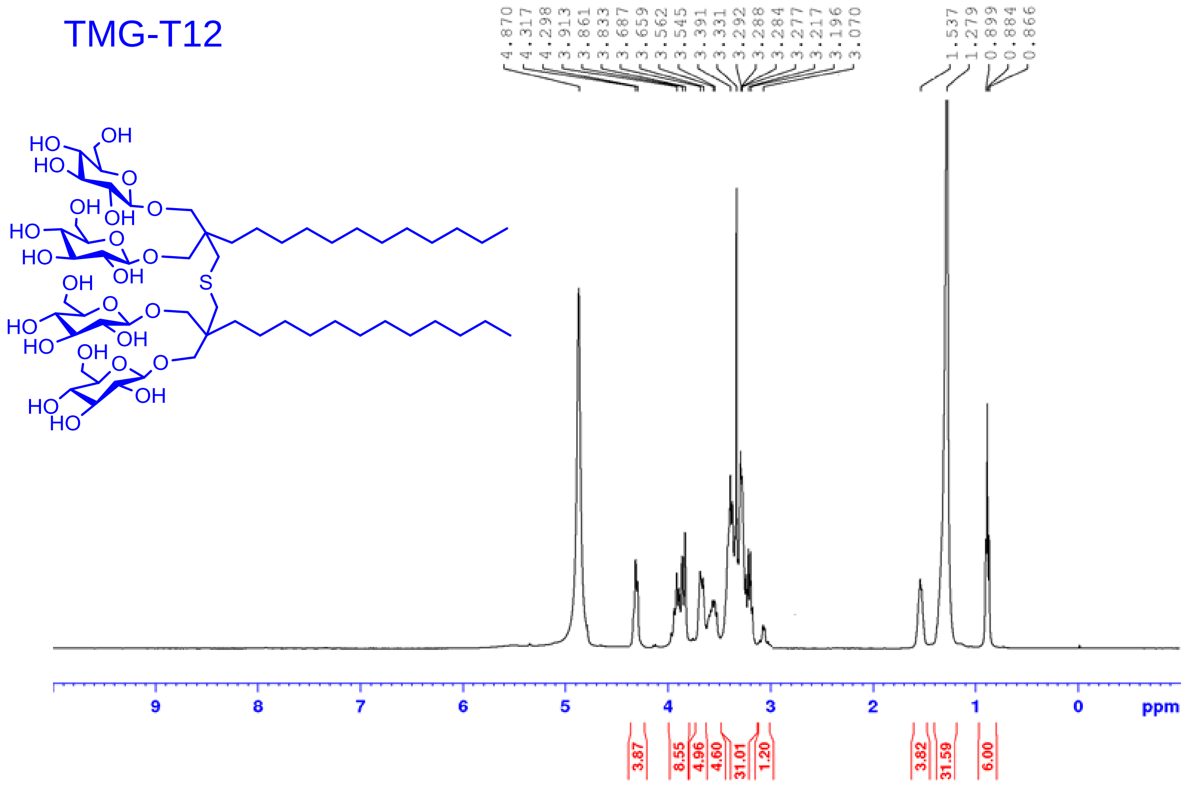

TMG-T13

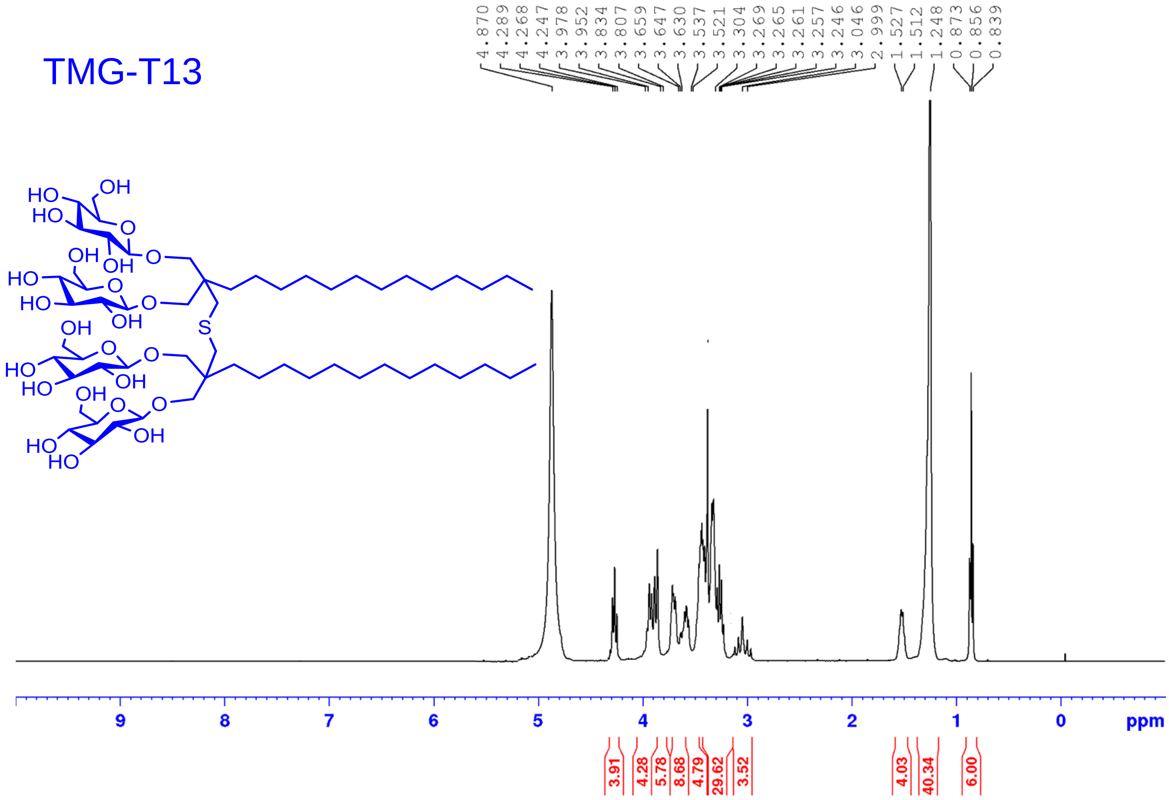

TMG-T14

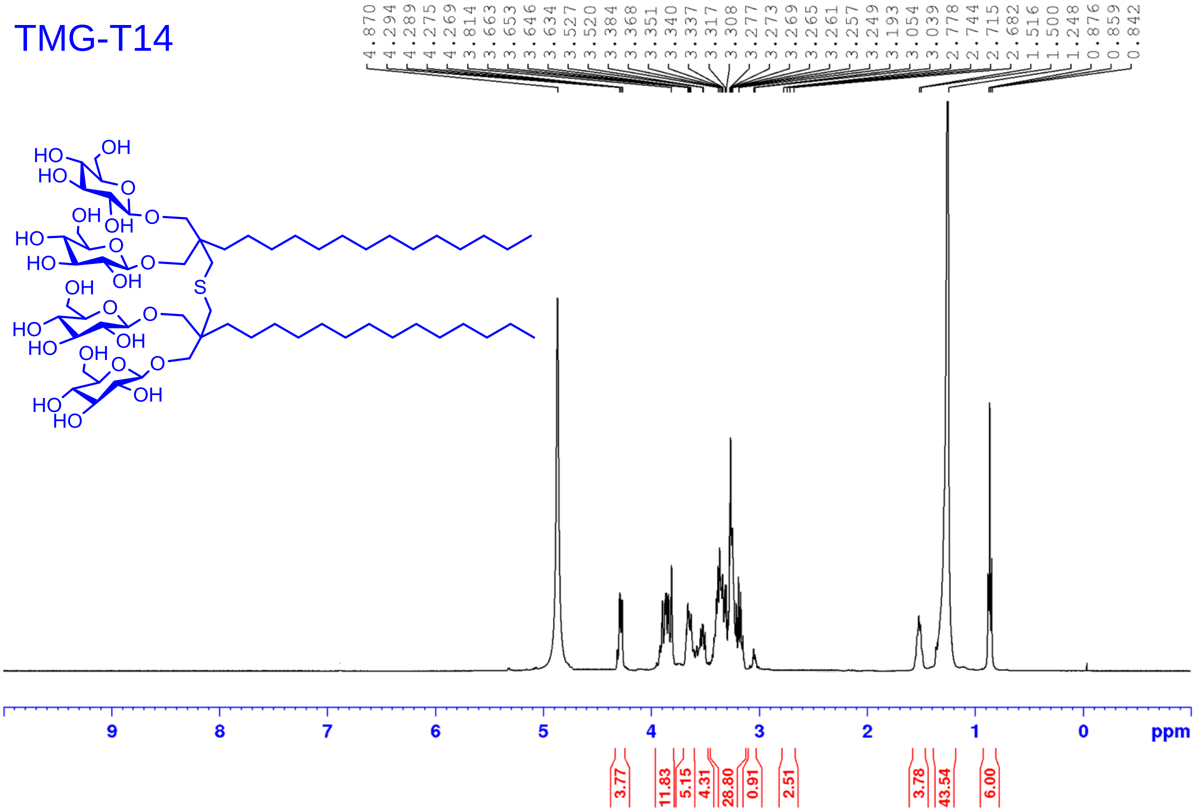

Supplement: Supplementary file 1 — Supplementary information [file 41598_2017_3809_MOESM1_ESM.pdf]
